# Polyphenols and miRNA interplay: a novel approach to combat apoptosis and inflammation in Alzheimer’s disease

**DOI:** 10.3389/fnagi.2025.1571563

**Published:** 2025-05-07

**Authors:** Minji Zhou, Xiu'e Pang

**Affiliations:** ^1^Chinese Medicine Hospital of Tiantai County, Taizhou, Zhejiang, China; ^2^Department of Neurology, Tiantai People's Hospital of Zhejiang Province, Tiantai Branch of Zhejiang Provincial People's Hospital, Hangzhou Medical College, Taizhou, Zhejiang, China

**Keywords:** inflammation, apoptosis, Alzheimer, miRNAs, polyphenol

## Abstract

Alzheimer’s disease (AD) is a neurodegenerative condition associated with aging. As the population ages, the incidence of AD has risen annually, making it the fourth leading cause of death, following cardiovascular disease, cancer, and stroke. The main pathological features of AD are now thought to include the accumulation of extracellular amyloid-β (Aβ) plaques, the formation of intracellular neurofibrillary tangles, and a reduction in synaptic connections in the cerebral cortex and hippocampus. Polyphenols help protect against AD by influencing Aβ metabolism. Research has shown that polyphenols are particularly effective in reducing inflammation and inhibiting tumor necrosis factor-activated TNF-κB activity, indicating their significant pharmacological activity. MicroRNAs (miRNAs) play a role in regulating miRNA stability and inhibiting protein expression after transcription. They are prevalent in brain tissue and can specifically influence neuronal growth and the formation of synapses. The expression levels of miRNAs in the brains of AD patients significantly differ from those in healthy individuals of the same age. miRNAs have been recognized as potential biological markers and therapeutic targets for the early diagnosis of AD. It is important to note that polyphenols can influence gene regulation by affecting the expression of various miRNAs, suggesting a potential link between polyphenols, AD, and miRNAs. This review examines whether polyphenols impact the expression of APP and Aβ. Additionally, we explored whether the effects of polyphenols on APP and Aβ are related to miRNAs.

## Introduction

AD was first identified in 1906 by German physician Alois Alzheimer, who noted plaque formation and neurofibrillary tangles in a 51-year-old female patient (Hippius and Neundörfer, 2003). The nervous system is especially susceptible to neurodegeneration due to its anatomical isolation, high energy demands for optimal functioning, and limited regenerative capacity. Furthermore, neurotransmission is the primary mechanism of biological communication in the nervous system (Hampel and Lau, 2020). Glial cells, especially astrocytes and microglia, play a crucial role in neurodegeneration by providing essential immunoregulatory neuroprotection (Vandenbark et al., 2021). AD is marked by a characteristic pathology involving the misfolding of specific proteins. Under physiological conditions, these proteins partially unfold and adopt an intramolecular cross-beta sheet conformation, resulting in the formation of toxic, insoluble fibrillar structures (Kundu et al., 2020). Age-related protein misfolding and aggregation have been linked to an increase in inflammatory mediators, such as proinflammatory cytokines, chemokines, nitric oxide (NO), and activated microglia. This inflammatory response ultimately contributes to neurodegeneration (Kundu et al., 2020).

miRNAs are endogenous, non-coding, single-stranded RNAs consisting of approximately 22 nucleotides, functioning as a class of gene regulators (O'Brien et al., 2018). Over 700 miRNAs have been cloned and sequenced in humans, and it is estimated that they regulate the post-transcriptional activity of about 30% of mammalian genes. Mature miRNAs typically downregulate gene expression, with their regulatory effect depending on the degree of complementarity with their target mRNAs (O'Brien et al., 2018). miRNAs that bind to the 3′ UTR of mRNA with imperfect complementarity inhibit protein translation, while those that bind with perfect complementarity can induce targeted mRNA cleavage. By altering the availability of mRNAs, miRNAs regulate a variety of cellular processes, including cell differentiation, growth, proliferation, and apoptosis (Gammell, 2007). Alterations in miRNA expression profiles are being extensively studied in various human diseases, including cancer, skeletal muscle disorders, and neurodegenerative diseases (Alkhazaali-Ali et al., 2024; Costa et al., 2020). Additionally, certain dietary components, such as polyphenols, have been reported to influence miRNA expression.

Polyphenols are the most common phytochemicals present in fruits, vegetables, and plant-based beverages (Zhang et al., 2022). They include a wide variety of compounds that are classified into several categories based on their chemical structures. These categories include phenolic acids (like hydroxybenzoic and hydroxycinnamic acids), flavonoids (which are divided into six subclasses: anthocyanins, flavanols, flavonols, flavones, flavanones, and isoflavones), as well as stilbenes, lignans, and curcuminoids (Singla et al., 2019). Most polyphenols are rarely present in foods as unconjugated aglycones; instead, they usually exist as conjugates with sugars or organic acids, or as polymers in the case of flavonoids (Singla et al., 2019). During absorption, dietary polyphenols undergo substantial metabolism by gut microbiota, followed by further processing in the intestine and liver. As a result, the main forms that enter the bloodstream and reach target tissues are conjugated metabolites, which differ chemically from the parent compounds found in plant foods (Ray and Mukherjee, 2021; Figueira et al., 2017). To date, there is no compelling evidence of long-term accumulation of water-soluble metabolites, even with regular consumption of high doses of polyphenols.

Research indicates that a high intake of fruits and vegetables rich in polyphenols may be linked to a reduced risk of various chronic conditions in humans, including inflammatory and metabolic disorders, cardiovascular disease, certain cancers, and neurodegenerative diseases (Rudrapal et al., 2022; Iqbal et al., 2023). These polyphenols have been closely linked to positive outcomes in various clinical, animal, and *in vitro* studies (Yang et al., 2021). Polyphenols, especially flavonoids, have been demonstrated to benefit cognitive function and help mitigate age-related neurodegenerative declines (Yang et al., 2021). Unlike many pharmaceutical compounds that target specific receptors or signaling pathways, polyphenols typically exert multitarget effects. Depending on the specific compounds, polyphenols may act through either nonspecific or specific mechanisms. This diversity in potential mechanisms explains the wide range of biological activities associated with polyphenols, including antiproliferative, antioxidant, anti-inflammatory, and pseudoestrogenic effects. Research has also provided new insights into the signaling pathways, transcription factors, and other potential regulators, such as miRNAs, that influence gene expression controlled by polyphenols.

Understanding how polyphenols affect neurodegenerative illnesses requires using cellular and animal models. By offering a controlled setting to evaluate the biological activity of polyphenols, especially their protective effects against oxidative stress and inflammation linked to neurodegeneration, these models aid in investigating the underlying molecular pathways (Rosado-Ramos et al., 2018). While *in vivo* investigations can highlight the intricacies of polyphenol metabolism and bioavailability in a living body, *in vitro* systems, such as 2D and 3D cell cultures, enable researchers to examine the direct effects of polyphenols on neuronal and glial cells (Mahmutović et al., 2024). However, there are drawbacks to these models as well. For example, the complex interactions and microenvironments present in biological tissues may not be accurately replicated in cell cultures, which could oversimplify the effects of polyphenols (Mahmutović et al., 2024). Furthermore, although animal models offer insightful information, they may differ from people in metabolism and reaction, which could compromise the findings’ generalizability. Therefore, even though cellular and animal models are crucial for studying polyphenols, it is essential to recognize their limits to appropriately extrapolate their applicability to neurodegenerative diseases in humans.

## Alzheimer diseases (AD)

**Figure 1:**
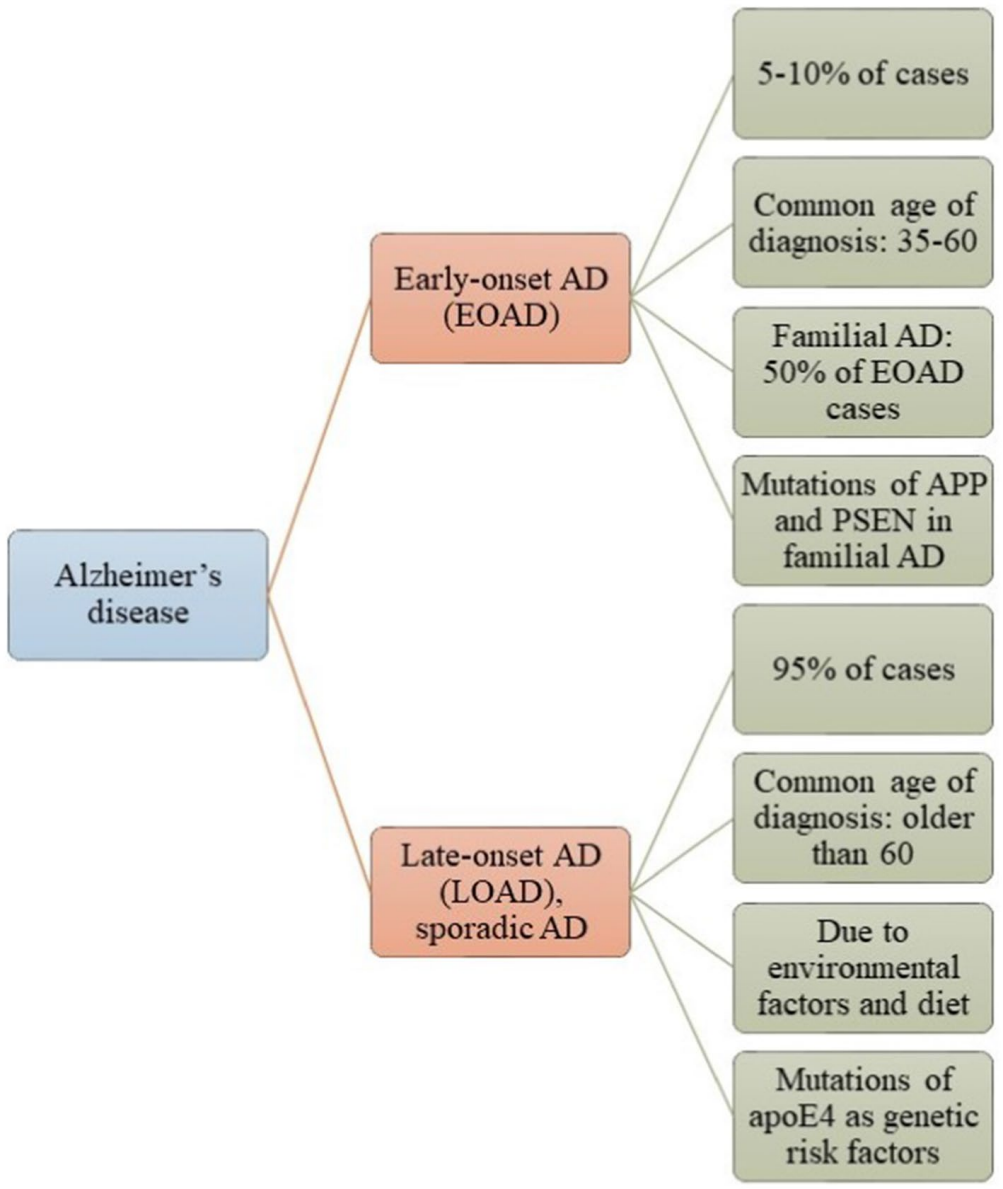
Alzheimer’s disease (AD) is classified into two categories: Early-Onset Alzheimer’s Disease (EOAD) and Late-Onset Alzheimer’s Disease (LOAD). Most patients with AD have LOAD, which typically manifests after the age of 65 and is influenced by environmental factors or genetic predispositions. In contrast, a smaller number of patients have EOAD, which is associated with mutations in the APP, PSEN, and Tau genes.

AD is the most common neurodegenerative disorder, impacting over 35 million people worldwide (Lanctôt et al., 2024). Aging is the primary risk factor, with the probability of developing AD doubling every 5 years after age 65 (Lanctôt et al., 2024). A substantial increase in prevalence is anticipated by mid-century (Lanctôt et al., 2024). AD is genetically complex, with over 100 rare mutations identified (Monteiro et al., 2023). The amyloid precursor protein (APP) is the most extensively studied due to its association with Aβ. Presenilins (PSENs) are critical elements in the pathogenesis of AD. They are fundamental to atypical aspartyl complexes that enable the cleavage of γ-secretase from the APP (Monteiro et al., 2023). Notably, presenilin 1 (PSEN1) is linked to early-onset AD (EOAD) (Bagaria et al., 2022) (Figure 1), which is characterized by mutations in APP, PSEN1, or presenilin 2 (PSEN2). The other variant is sporadic or late-onset AD (LOAD) (Figure 1), which is non-dominant and primarily associated with the Apolipoprotein ε4 (APOE4) allele, responsible for approximately 95% of AD cases (Raulin et al., 2022). The APOE ε4 allele is linked to an increased risk of AD in individuals with Down syndrome, as well as those who have experienced traumatic brain injury or stroke (Raulin et al., 2022). The APOE4 allele is also associated with metabolic risks due to its involvement in cholesterol and triglyceride metabolism (Yang et al., 2023). APOE interacts with specific receptors, such as the LDL receptor-related protein 1 (LRP1) and the very-low-density lipoprotein receptor (VLDLR), aiding in the clearance of chylomicron and VLDL residues from circulation. This mechanism is crucial for the normal catabolism of triglyceride-rich lipoproteins (Yang et al., 2023).

Several hypotheses have been proposed to explain the origins and progression of AD, including the cholinergic, Tau, Aβ, oxidative stress, vascular, and inflammation hypotheses (Cáceres et al., 2023; Twarowski and Herbet, 2023). Among these, the amyloid cascade hypothesis suggests that neurodegeneration results from a sequence of events initiated by the improper processing of APP, leading to the accumulation of amyloid-beta (Aβ) and subsequent pathological changes in the brain (Cáceres et al., 2023). The cholinergic theory posits that a deficiency in acetylcholine—a neurotransmitter critical for memory and learning—contributes to the cognitive decline observed in AD (Cáceres et al., 2023). In contrast, the oxidative stress hypothesis argues that oxidative damage to cells, caused by free radicals, plays a significant role in neuronal injury and degeneration in AD. The Tau hypothesis centers on the role of tau protein, which can become hyperphosphorylated, leading to the formation of neurofibrillary tangles (Cáceres et al., 2023). This hypothesis suggests that tau pathology is central to the progression of AD (Cáceres et al., 2023). On the other hand, the vascular theory emphasizes the significance of cerebrovascular health in AD, proposing that impairments in blood flow and vascular function may contribute to or exacerbate the disease. The inflammation theory in AD suggests that inflammatory processes in the brain share similarities with those in other systems of the body (Twarowski and Herbet, 2023). This theory posits that inflammatory regulators are often elevated in regions where AD pathology is pronounced, indicating a potential link between neuroinflammation and the progression of the disease (Twarowski and Herbet, 2023). Activated microglia and reactive astrocytes tend to cluster around fibrillar plaques in AD (Serrano-Pozo et al., 2013; Wu and Eisel, 2023). These chronically activated microglia release chemokines, initiating a cascade of harmful cytokines, including interleukins such as TNF-α, IL-1, and IL-6 (Serrano-Pozo et al., 2013; Wu and Eisel, 2023). Microglia possess receptors for advanced glycation end products that bind to Aβ, which further increases the production of cytokines, glutamate, and NO (Wu and Eisel, 2023). Additionally, microglia release proteins like alpha-2-macroglobulin (A2M), C-reactive protein (CRP), and alpha-1-antichymotrypsin (ACT), all of which can exacerbate the pathology of AD (Wu and Eisel, 2023).

## Neuroinflammation in Alzheimer diseases

Neuroinflammation plays several roles in the brain (Adamu et al., 2024). Typically, inflammation serves as a defense mechanism, helping to remove pathogens, cellular debris, misfolded proteins, and other harmful stimuli to preserve or restore tissue integrity (Adamu et al., 2024). However, when neuroinflammation becomes uncontrolled, it can lead to neuronal degeneration and disrupt the blood–brain barrier (BBB) (Adamu et al., 2024). This detrimental process is characterized by the release of pro-inflammatory cytokines, chemokines, and small-molecule messengers, primarily produced by activated microglia and astrocytes (Adamu et al., 2024). The innate immune response to viral infections or traumatic brain injuries activates glial cells, such as microglia and astrocytes, which are crucial for regulating immunity and maintaining homeostasis in the central nervous system (CNS) (Jorgačevski and Potokar, 2023; Ismail et al., 2024). This regulation involves a series of interactions between microglia and astrocytes, which can lead to neurodegeneration at multiple molecular levels. These processes encompass cell death mediation, synaptic remodeling, and immune signaling regulation, including mechanisms like apoptosis and autophagy (Adamu et al., 2024). The complement system plays a crucial role in maintaining the balance between acute and chronic neuroinflammation within the CNS microenvironment (Chen et al., 2022). Understanding this balance is key to comprehending CNS immunoregulation and its implications for neurodegenerative diseases (Chen et al., 2022). Neuroinflammation is generally beneficial for managing external stressors effectively. However, it can become detrimental when the immune response is prolonged, particularly due to immunosenescent aging (Adamu et al., 2024). This chronic inflammation leads to dysregulation of immune signaling, which can ultimately contribute to the development of neurodegenerative diseases (Adamu et al., 2024). Microglia are the resident immune cells of the CNS and function similarly to specialized macrophages (Perry and Teeling, 2013). Unlike other glial cells, such as astrocytes, microglia originate from hematopoietic cells in the yolk sac (Perry and Teeling, 2013). They serve as the primary responders to infections and injuries, playing a crucial role in the immune defense and maintenance of CNS homeostasis (Perry and Teeling, 2013). Microglia perform a variety of functions, including regulating programmed cell death in neurons, removing excess synapses during development, and promoting the formation of neurites (Perry and Teeling, 2013). In a well-functioning brain, microglia exist in an inactive state, where they remain stationary while their cellular processes extend and retract (Adamu et al., 2024; Perry and Teeling, 2013). This dynamic behavior allows them to monitor their surroundings and communicate effectively with neurons and other glial cells (Adamu et al., 2024; Perry and Teeling, 2013). Microglia are more complex than previously thought and are highly diverse cells. Both extrinsic (pathogens, diet, microbiome, etc.) and internal (species, sex, genetic background, etc.) factors affect the states of microglia (Paolicelli et al., 2022; Gao et al., 2023). Numerous neurodegenerative illnesses have been linked to reactive microglia, which are microglia that undergo morphological, molecular, and functional remodeling in response to brain challenges (such as amyloid β [Aβ] or α-synuclein [α-syn] deposits, infected, injured, or degenerating neurons). These reactive microglia have been found to exhibit significant levels of spatial and temporal variability as well as distinct disease-related signals that do not conform to the standard M1/M2 classification in neurodegenerative illnesses, thanks to recent developments in scRNA-seq and snRNA-seq technologies (Gao et al., 2023; Keren-Shaul et al., 2017; Friedman et al., 2018; Gerrits et al., 2021). For example, scRNA-seq research has discovered disease-associated microglia (DAMs), a particular microglial response state, in AD patient specimens and mice models (Gao et al., 2023; Keren-Shaul et al., 2017; Friedman et al., 2018; Mathys et al., 2017). Interestingly, DAMs were found close to Aβ plaques and helped to remove β-amyloid. Furthermore, different microglia signatures linked to tau and Aβ have been found in AD patients. To effectively treat AD and other neurodegenerative illnesses, it is essential to identify disease-specific microglial states and investigate variables influencing them. These findings imply that microglia exhibit plasticity when responding to different pathologies. Microglial activation in AD is thought to be primarily triggered by the presence of Aβ plaques (Azargoonjahromi, 2024). Activated microglia (DAMs) respond to Aβ by migrating toward these plaques and attempting to phagocytose the Aβ (Azargoonjahromi, 2024). While several studies have demonstrated that activated microglia can phagocytose Aβ, prolonged exposure leads to microglial enlargement and a diminished capacity to effectively process Aβ (Azargoonjahromi, 2024). This impaired response contributes to the accumulation of Aβ and exacerbates neuroinflammation, ultimately playing a role in the progression of AD (Azargoonjahromi, 2024). In the early stages of AD pathogenesis, the activated immune response facilitates the clearance of Aβ and has shown positive effects on AD-related pathologies in animal models (Chen and Holtzman, 2022). However, prolonged activation of the immune response can worsen AD pathology (Chen and Holtzman, 2022). This is likely due to the sustained activation of microglia in a feedback loop known as reactive microgliosis, where continuously activated microglia contribute to chronic inflammation and neuronal damage, further exacerbating the disease progression (Chen and Holtzman, 2022). Balancing the immune response is crucial for mitigating AD pathology (Chen and Holtzman, 2022). This prolonged activation leads to an accumulation of Aβ and persistent signaling of pro-inflammatory cytokines, which begins to damage neurons (Chen and Holtzman, 2022). Additionally, the sustained activation reduces the efficiency of microglia in binding and phagocytosing Aβ, along with a decline in the activity of Aβ-degrading enzymes (Chen and Holtzman, 2022; Merighi et al., 2022). This creates a vicious cycle where the inability to clear Aβ contributes to further neuronal damage and inflammation, ultimately worsening AD pathology. Consequently, this results in a decreased ability to break down Aβ plaques, while the capacity of microglia to produce pro-inflammatory cytokines remains unchanged (Merighi et al., 2022). This highlights a unique aspect of AD pathogenesis: the overall clearance of Aβ is impaired even as immune activation continues concurrently. This paradox creates an environment where inflammation persists without effective resolution, further contributing to neuronal damage and the progression of the disease. The persistent release of pro-inflammatory cytokines and related neurotoxins from microglia intensifies neuroinflammation and contributes to neurodegeneration, which in turn leads to the activation of additional microglia (Merighi et al., 2022). As these activated microglia attempt to clear Aβ, they release a range of pro-inflammatory cytokines that attract even more microglia to the plaques (Merighi et al., 2022). This results in a distinctive halo of activated microglia surrounding the Aβ plaques, creating a feedback loop that exacerbates inflammation and neuronal damage, further advancing the pathology of AD (Merighi et al., 2022). As microglia lose their effectiveness in clearing Aβ, peripheral macrophages may be recruited to areas of Aβ plaque deposition to assist with Aβ clearance (Merighi et al., 2022; Jay et al., 2015). However, the influx of these peripheral macrophages into the brain likely intensifies the effects of sustained inflammation, further worsening AD pathology. This recruitment can exacerbate neuroinflammation and contribute to the overall neurodegenerative process, highlighting the complex interplay between central and peripheral immune responses in AD.

The snRNA-seq study of human astrocytes across five brain regions by Serrano-Pozo et al. (2024) revealed several key findings related to the aging process and AD progression. Significant regional differences in the astrocyte transcriptome were observed, particularly in the entorhinal cortex (EC) and primary visual cortex (V1), with the EC—especially layer II—being the first area affected by neurofibrillary tangles (NFTs) in AD (Serrano-Pozo et al., 2024). An EC-specific astrocyte signature was identified, characterized by the upregulation of genes such as APP and APOE, alongside the downregulation of SLC1A2 (glutamate transporter) and MAPT (tau protein), suggesting a role for these genes in the vulnerability of EC astrocytes to AD (Serrano-Pozo et al., 2024). The study also tracked astrocyte gene expression along a spatial axis corresponding to the typical progression of AD, identifying distinct gene sets that followed specific spatial trajectories influenced by local levels of Aβ and pTau. As AD pathology progressed, astrocytes exhibited altered expression of genes related to synaptic function, energy metabolism, and neuroinflammation, with chronic exposure to Aβ and pTau linked to energy deficits in astrocytes, thereby contributing to neurodegeneration (Serrano-Pozo et al., 2024). Temporal dynamics of astrocyte responses revealed successive waves of transcriptomic changes associated with AD severity, showing that intermediate stages led to the upregulation of trophic factors and neuroinflammatory genes. In contrast, late-stage responses were dominated by genes involved in proteostasis and energy metabolism (Serrano-Pozo et al., 2024). A transitional state between homeostatic and reactive astrocytes, termed astIM, was identified, indicating that astrocytes can dynamically change states in response to their microenvironment (Serrano-Pozo et al., 2024). In end-stage AD (Braak stage VI), many genes associated with reactive astrocytes were downregulated, suggesting exhaustion of astrocytic responses following chronic exposure to pathological conditions (Serrano-Pozo et al., 2024).

## Apoptosis in Alzheimer diseases

**Figure 2:**
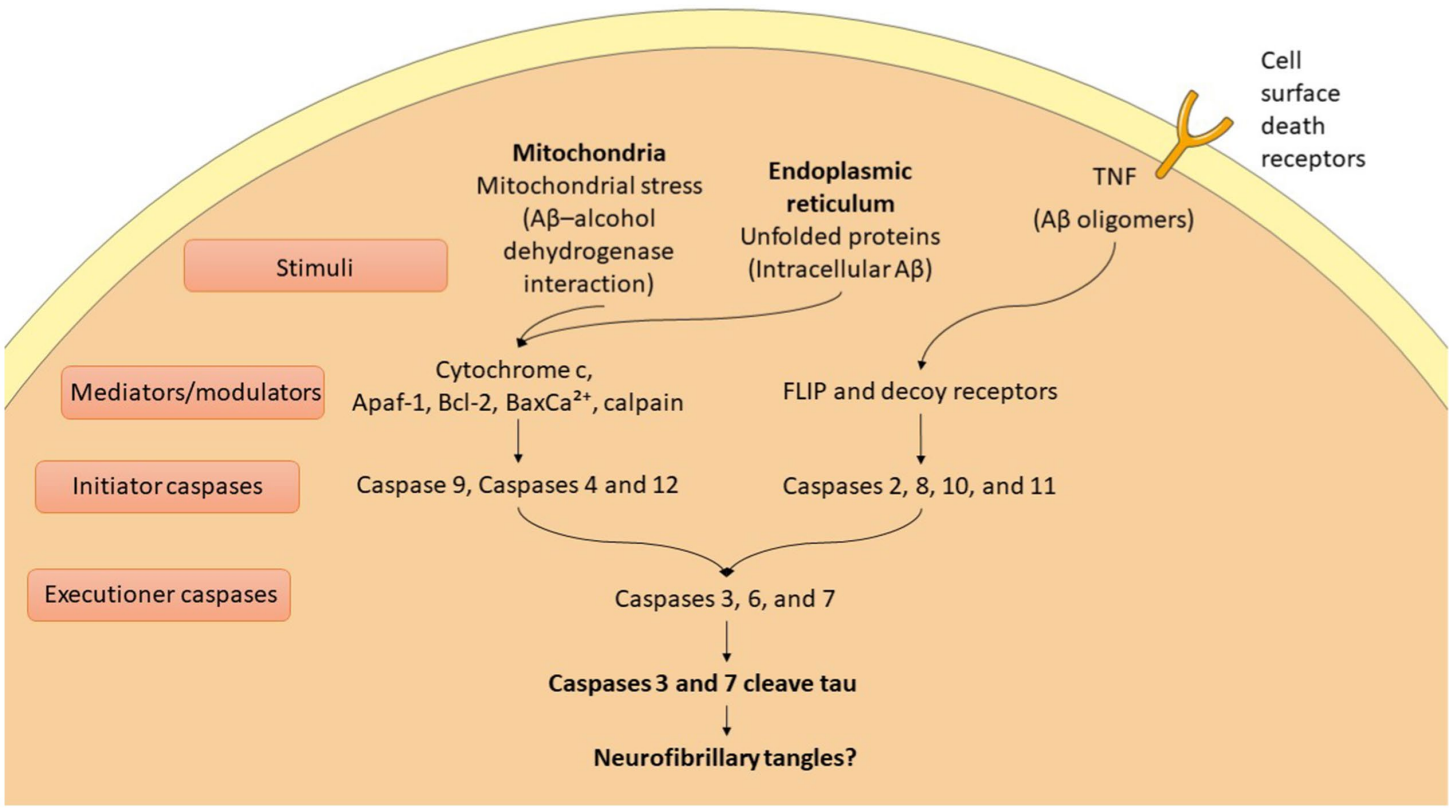
Apoptotic mechanisms in Alzheimer diseases (AD).

AD is characterized by the buildup of hyperphosphorylated tau protein and Aβ, leading to synaptic loss and neuronal apoptosis (Rajmohan and Reddy, 2017). The accumulation of Aβ and tau activates apoptotic pathways that ultimately result in neuronal death (Kumari et al., 2023; Zhang et al., 2021). This neuronal loss plays a critical role in the cognitive decline and other symptoms associated with AD, highlighting the interplay between these pathological hallmarks and their impact on brain function (Kamatham et al., 2024). In AD, apoptosis is regulated through both extrinsic and intrinsic pathways, involving the activation of various proteins (Kumari et al., 2023) (Figure 2). Key players include members of the Bcl-2 family, such as Bax, Bad, Bid, Bcl-XS, and Bcl-XL, which help mediate the apoptotic process (Kumari et al., 2023; Qian et al., 2022). Additionally, caspases play a crucial role and are categorized into initiator, effector, and inflammatory caspases (Kumari et al., 2023; Van Opdenbosch and Lamkanfi, 2019). This intricate network of proteins and enzymes contributes to the neuronal death observed in AD, further amplifying the disease’s progression and associated symptoms. This process unfolds through a series of events that ultimately lead to cell disintegration. Apoptotic mechanisms interact with various trophic factors and signaling pathways, influencing neuronal health and survival (Kumari et al., 2023). Notably, the Ras–ERK pathway plays a significant role in regulating both cell cycle progression and apoptosis (Kumari et al., 2023). Dysregulation of this pathway can contribute to the imbalance between cell survival and death, exacerbating the neurodegenerative processes in AD. In AD, the upregulation of the JNK pathway leads to reduced levels of anti-apoptotic proteins, promoting cell death (Kumari et al., 2023). The JAK–STAT pathway also initiates caspase-3 mediated apoptosis, contributing to neurodegeneration. Additionally, the PI3K/Akt/mTOR pathway plays a crucial role in maintaining the balance between autophagy and apoptosis; any dysregulation in this pathway can result in increased apoptosis (Kumari et al., 2023). Furthermore, glycogen synthase kinase-3β (GSK-3β) activates pro-apoptotic factors, further disrupting the regulation of apoptosis (Kumari et al., 2023). Together, these pathways highlight the complex interplay of signaling mechanisms that drive neuronal loss in AD.

## Role miRNAs in Alzheimer diseases

**Figure 3:**
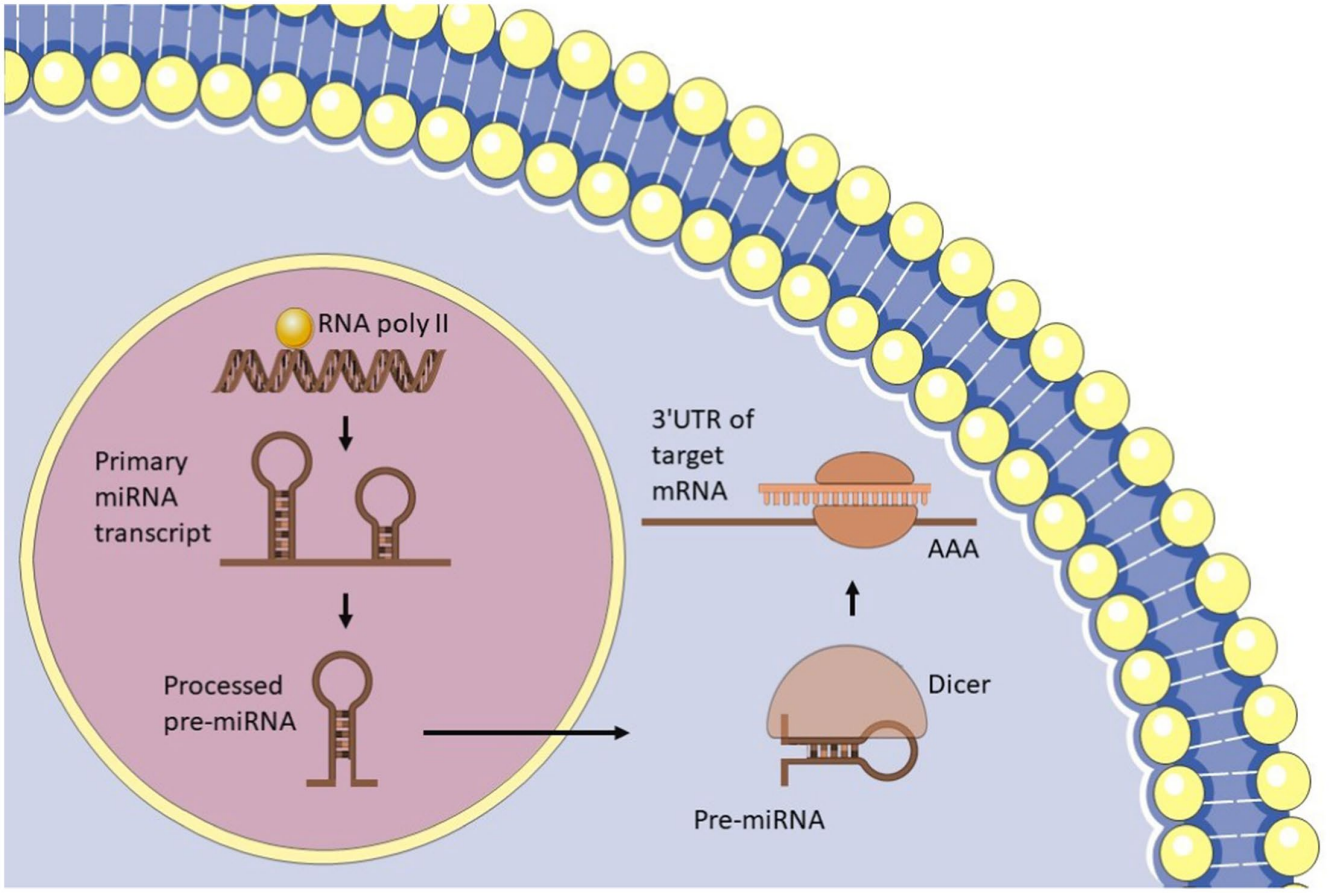
microRNA biogenesis.

miRNAs are small, non-coding RNA molecules, typically 20–24 nucleotides in length, that play a crucial role in regulating gene expression in eukaryotic cells (Bofill-De Ros and Vang Ørom, 2024). They exert their regulatory effects by binding to the messenger RNA (mRNA) of target genes, influencing both transcription and translation. This interaction can lead to the degradation of mRNA or inhibit its translation, thereby modulating the expression levels of various proteins and contributing to numerous cellular processes, including development, differentiation, and responses to stress. Additionally, miRNAs are essential for various cellular functions, including cell proliferation, differentiation, apoptosis, metabolism, and immune response. The biogenesis of miRNAs is an intricate process that involves several steps, beginning with transcription in the nucleus (Bofill-De Ros and Vang Ørom, 2024) (Figure 3). This process produces primary miRNA (pri-miRNA), which is then processed by the Drosha enzyme to form precursor miRNA (pre-miRNA) (Gregory et al., 2004). The pre-miRNA is exported to the cytoplasm, where it is further processed by the Dicer enzyme to generate mature miRNA (Bartel, 2018). These mature miRNAs then participate in regulating gene expression by binding to target mRNAs, highlighting their critical role in cellular regulation and homeostasis. The six key processes that influence miRNA levels are as follows: the transcription of the primary miRNA (pri-miRNA) from DNA; the processing of this primary transcript into precursor miRNAs (pre-miRNA); the export of pre-miRNA to the cytoplasm; the further processing of pre-miRNA into mature miRNA; the recognition, inhibition, or cleavage of target mRNAs; and the mechanisms that control the stability and degradation of mature miRNAs (Bofill-De Ros and Vang Ørom, 2024). Initially, RNA polymerase II transcribes the pri-miRNA from DNA (Georgakilas et al., 2014). This primary sequence contains the pre-miRNA, which adopts a hairpin structure. The Microprocessor complex, consisting of the RNase III enzyme DROSHA and its cofactor DGCR8, recognizes and cleaves this structure, resulting in the formation of a processed hairpin-shaped pre-miRNA (Gregory et al., 2004). The Exportin 5 (XPO5) protein transports the pre-miRNA to the cytoplasm (Yi et al., 2003), where it is further processed by the RNase III enzyme DICER, in conjunction with its cofactor TRBP (Bartel, 2018). This processing produces a double-stranded RNA duplex that consists of the mature miRNA and its complementary passenger strand. The duplex is unwound, allowing the mature miRNA to be incorporated into the RNA-induced silencing complex (RISC). The RISC/miRNA complex subsequently binds to target mRNAs, resulting in the repression of their translation or facilitating their degradation (McGeary et al., 2019).

miRNAs have been linked to the progression of several diseases, including cancer, cardiovascular diseases, and neurological disorders such as AD (Li et al., 2024; Elsakka et al., 2023; Mok et al., 2024). They are known to regulate essential processes, including inflammation, differentiation, proliferation, apoptosis, immune responses, and neurodegeneration (Li et al., 2023a; Das and Rao, 2022; Galagali and Kim, 2020; Xu et al., 2020; Gaál, 2024). In AD, changes in miRNA expression levels are linked to key pathological features, including tau protein phosphorylation and aggregation, mitochondrial dysfunction, and the production of Aβ peptides (Praticò, 2020; Catanesi et al., 2020; Amakiri et al., 2019). These alterations in miRNA activity contribute to neuronal dysfunction and cell death, significantly influencing the progression of AD. Studies on miRNAs in AD provide valuable insights into the molecular mechanisms underlying the condition and highlight the potential for using miRNAs as biomarkers for diagnosis and as targets for therapeutic intervention. In conclusion, miRNAs are crucial regulators of gene expression, influencing a wide array of biological processes and disease pathologies. Abnormal miRNA expression is closely linked to the development and progression of various diseases, including AD.

## Regulation of apoptosis and neuroinflammation by miRNAs in animal models of Alzheimer’s disease

miRNAs regulate various pathways related to neuronal function, apoptosis, inflammation, and survival. Research on miRNAs offers a valuable new perspective for exploring the mechanisms involved in the development of AD. Animal studies concerning miRNAs in AD are summarized in Table 1. In this article, we will discuss the role of microRNAs that are involved in apoptosis and inflammation in animal models of AD (Table 2).

**Table 1:** Overview of miRNAs associated with Alzheimer diseases.

miRNAs	Expression	AD-Relevant targets	Animal model	Outcomes	Ref.
miRNA-128	↑	PPARγ	3 × Tg-AD, 3 × Tg-AD-KO, WT C57BL/6	Cerebral cortex Aβ production↑, AD-like performances↑, amyloid plaque formation and Aβ generation ↑, the amyloidogenic processing of APP↑, neuroinflammation↑	Liu et al. (2019)
miRNA-222	↓	p27Kip^1^	APPswe/PSΔE9 mice	N/A	Wang et al. (2015)
miRNA-574	↑	SYN and Nrn1	APP/PS1 mice	Synaptic loss and cognitive deficits↑	Li et al. (2015)
miRNA-214–3p	↓	LC3βII and Beclin1	SAMP8 mice	Cognition defects↑, autophagy↑, number of GFP-LC3-positive autophagosome vesicles↑, caspase-mediated apoptosis↑	Zhang Y. et al. (2016)
miRNA-206	↑	BDNF	APP/PS1 transgenic mice	Cell death↑	Tian et al. (2014)
miRNA-181c	↓	Crmp2	SAMP8 mice	N/A	Zhou et al. (2016)
miRNA-181	↑	c-Fos and SIRT-1	3xTg-AD	Synaptic plasticity↑, memory processing↓	Rodriguez-Ortiz et al. (2014)
miRNA-146a	↑	ROCK1	5xFAD	Tau hyperphosphorylation↑, memory function↓	Wang et al. (2016)
miRNA-132	↓	ITPKB	APPPS1 mice	Aβ generation and TAU phosphorylation↑	Salta et al. (2016)
miRNA-132/212	↓	Tau, Mapk, Sirt1	3xTg-AD mice	Aβ production and senile plaque deposition↑	Hernandez-Rapp et al. (2016)
miRNA-188-5p	↓	CREB	5XFAD transgenic mice	Dendritic spine density and basal synaptic transmission↑, synaptic dysfunction↑, memory deficits↓	Lee et al. (2016)
miRNA-139	↑	cannabinoid receptor type 2 (CB2)	SAMP8 mice	Acquisition and retention of spatial memory↓, novel object recognition and contextual fear conditioning↓, responses to pro-inflammatory stimuli↓	Tang et al. (2017)
miRNA-125b	↓	-	APP/PS1 transgenic mouse model	Cognitive abilities↓, Neuronal loss and synaptic dysfunction↑	Hong et al. (2017)
miRNA-302/367	↓	NeuN	STZ-Induced C57/BL6J mice	Learning and memory↓, astrocyte reactivation↓	Ghasemi-Kasman et al. (2018)
miRNA-34a	↑	Amyloidogenic processing of APP	APP/PS1 mice	Cognitive deficits↑, Aβ accumulation↑	Jian et al. (2017)
miRNA-34a	↑	NMDA receptors	APP/PSEN1 mice	Synaptic deficits↑	Xu et al. (2018)
miRNA-34a	↑	Metabotropic glutamate receptor 7 (GRM7)	3xTg-AD mice	Anxiety-like behaviors↑	Zhang Y.L. et al. (2016)
miRNA-10a-5p, miRNA-142a-5p, miRNA-146a-5p, miRNA-155-5p, miRNA-211-5p, and miRNA-455-5p	↑		APPtg (APPswe/PS1L166P) and TAUtg (THY-Tau22) mice	N/A	Sierksma et al. (2018)
miRNA-137	↓	CACNA1C	APP/PS1 transgenic mice	Spatial learning and memory, serum, hippocampus, and cerebral cortex levels of Aβ1-40 and Aβ1-42↑, hyperphosphorylation of tau↑	Jiang et al. (2018)
miRNA-29c	↓	BACE1 and Aβ	SAMR1 and SAMP8 mice	Learning and memory behaviors↓	Yang et al. (2015)
miRNA-29c	↑	NAV3	APPswe/PSΔE9 transgenic mice	Cognitive defect↓	Zong et al. (2015)
miRNA-200a-3p	↑	SIRT1	APPswe/PS ΔE9 transgenic mice	β-Amyloid-induced neuronal apoptosis↑	Zhang et al. (2017)
miRNA-135a, -200b and -429	↓	BACE-1, APP	APP/PS1 transgenic mice	N/A	Liu et al. (2014)
miRNA-200 family (miRNA-200a, -141, -429, -200b, -200c)	↑	Ribosomal protein S6 kinase B1 (S6K1)	Tg2576 transgenic mice	Aβ secretion↑, Aβ-induced cognitive impairment↑	Higaki et al. (2018)
miRNA-155	↑	–	C57/BL6 WT mice	Internalization and catabolism of extracellular fAβ1-42↓	Aloi et al. (2021)
miRNA-155	↑	Tfeb	APP/PS1 mice, 5XFAD mouse	insoluble Aβ1-42↑, Anti-Inflammatory Responses↓	Aloi et al. (2023)
miRNA-155	↑	SOCS-1	3xTg AD	Activation of microglia and astrocytes↑, IL-6 and IFN-β↑	Guedes et al. (2014)
miRNA-155	↑	PI3K-Akt signaling	APPSWE/PS1L166P AD	Retinal inflammation↑, IL-8 and Spp1 levels↑	Shi et al. (2022)
miRNA-155	↑	–	APP/PS1 mice	Aβ oligomer levels and amyloid plaque density↓	Readhead et al. (2018)
miRNA-146a	↑	CFH, IRAK-1, tetraspanin-12 (TSPAN12)	5XFAD mouse model	Anti-Inflammatory Responses↑	Li et al. (2011)
miRNA-135a-5p	↓	Rock2/Add1	AD model mice	Memory impairments↑, synaptic disorder↑	Zheng et al. (2021)
miRNA-132	↓	DOCK1, EPHB3, BTG2, CAMK1, RAC1	APP/PS1 mice	Hippocampal neurogenesis↓, memory deficits↑, proliferation and differentiation deficits of adult neural precursors↑	Walgrave et al. (2021)
miRNA-98-5p	↑	α7 nAChR	APP/PS1 mice	Cognitive deficits↑, Aβ burden↑, neuroinflammation↑	Song C. et al. (2021)
miRNA-31	↓	APP and BACE1	3xTg-AD	Cognitive Deficits↑, Extracellular and Intracellular Aβ Deposition↑	Barros-Viegas et al. (2020)
miRNA-455-5p	↑	CPEB1	APP/PS1 mice	Deficits in synaptic plasticity and memory↑	Xiao et al. (2021)
miRNA-155-5p	↑	SKP2/IKKβ	APP.PS1 mice	Cognitive impairment↑, neuron regeneration↓, Aβ deposition↑	Wang et al. (2022)
miRNA-17	↑	Microglial ASC, CCL3/MIP1α	5XFAD mouse model	Autophagy↓, Aβ accumulation↑, and inflammatory cytokine production↑, Aβ burdens↑, immune cell activation pathways↑, spatial memory↓, anxiety behavior ↑	Badr et al. (2024)
miRNA-369-5p	↑	GluA1 and GluA2	3xTg-AD mice	OLM impairment↑	Liu and Xiang (2022)
miRNA-451a	↓	BACE1	APP/PS1 transgenic mice	Long-term spatial memory↑, depression-like behavior↑	Feng et al. (2023)
miRNA-181a	↑	AMPA receptor subunits GluA2 and GluA1	3xTg-AD model	Disruption plasticity↑, Aβo-induced deficits in plasticity↑	Rodriguez-Ortiz et al. (2020)
miRNA-223-3p	↓	NLRP3/GSDMD	STZ-induced sporadic mouse model of AD (sAD)	Neuroinflammation↑, amyloid deposition↑, neuronal apoptosis↑, cognitive dysfunction↑	Lin L. et al. (2024)
miRNA-128	↓	GSK3β, APPBP2, mTOR	5XFAD mice	Tau phosphorylation and Aβ secretion ↑, Aβ levels↑, cognitive deficits↑, plaque burden↑	Li et al. (2023b)
miRNA-134-5p	↑	CREB-1 and BDNF	C57BL/6 mice	Aβ (1–42)-induced deficit↑, impairment of synaptic tagging and capture↑	Baby et al. (2020)
miRNA-223	↓	YB-1	APP/PS1 mice	Nerve damage↑, neuroinflammation ↑ cognitive impairments↑	Wei et al. (2024)
miRNA-29a	↑	Plxna1, Dio2 and Wdfy1	5 × FAD and WT mice	Cognitive Decline↓, Beta-Amyloid Deposition↑	Mei et al. (2024)
miRNA-140	↑	PINK1	Sprague–Dawley rats	Autophagy↓, ROS and mitochondrial dysfunction↑, spatial learning and memory ability↓	Liang et al. (2021)
miRNA-137-5p	↓	USP30	Male C57BL/6 J	Spatial memory and cognition↓, Aβ1–42-induced apoptosis↑	Jiang et al. (2023)
miRNA-33	↑	ABCA1-APOE	APP/PS1 mice	Insoluble Aβ peptides and plaque deposition↑, astrocyte and microglial activation↑	Tate et al. (2024)
miRNA-196b-5p, miRNA-339-3p, miRNA-34a-5p, miRNA-376b-3p, miRNA-677-5p, and miRNA-721	↓ or ↑	Tgfb, Notch1	5XFAD mouse	N/A	Song Z. et al. (2021)
miRNA-483-3p	↓	XPO1	Sprague–Dawley rats injected with homocysteine (Hcy)	Learning and memory abilities↓	Luo et al. (2022)
miRNA-29b-2	↓	PSEN-1	3xTg-AD mouse	Production of amyloid proteins↑, cognitive impairment↑	Lin E.Y. et al. (2024)
miRNA-204-3p	↓	Nox4	APP/PS1 mice	Memory and synaptic deficits↑, amyloid levels and oxidative stress↑	Tao et al. (2021)
miRNA-200c	↓	4–3-3γ, p-GSK-3β	5xFAD mouse	Tau hyperphosphorylation↑, cognitive impairment↑	Park et al. (2022)
miRNA-1271	↓	Grb2 and NOX4	APP/PS1 mice, B6C3-Tg (APPswe, PSEN1dE9/) 85Dbo/J mice	Cytoskeleton disruption↑	Majumder et al. (2021)
miRNA-146a and miRNA-34a	↑	ROR1 and Vimentin	APP/PS1 mice	Cytoskeleton disruption↑	Chanda et al. (2021)
miRNA-29c-3p	↓	BACE1	STZ-induced -sAD	Apoptosis↑ and Viability of Aβ-Induced PC12 Cells↓	Cao et al. (2021)
miRNA-142	↑	pCaMKII and BAI3	APP/PS1 transgenic mice	Impairment of spatial learning and memory↑, apoptosis↑	Fu et al. (2021)
miRNA-146a	↓	FoxO6, Nkd2, Cd4 and Stpg1	APP/PS1 transgenic mice	Neuroinflammation↑ and in the microglial phenotype↓, Aβ levels and amyloid plaques↑, plaque-associated neuritic pathology↑, neuronal loss↑, microglial phagocytosis↓, apoptosis↑	Liang et al. (2021)
miRNA-132-3p	↓	FOXO3a-PPM1F	APP/PS1 mice	N/A	Fu et al. (2022)
miRNA-425	↓	PI3K-AKT	APP/PS1 mouse	Amyloidogenic APP processing↑, reactive gliosis↑, neuron loss↑, impaired synaptic plasticity and cognitive impairment↑, changes global RNA expression profiles associated with AD pathological alterations	Hu Y.B. et al. (2021)
miRNA-199a	↑	Neuritin	APP/PS1 mice	N/A	Song et al. (2020)
miRNA-27a	↑	SOX8/ β-catenin	Aβ25-35-induced rat AD mode	N/A	Hu Y. et al. (2021)
miRNA-96-5p	↑	Foxo1	AD mice	Pathological changes in the neurons of AD mice↑	Wu et al. (2020)
miRNA-130a-3p	↓	DAPK1	APP/PS1 mice	Cognitive Function↓	Wang et al. (2021)
miRNA-206-3p	↑	STIM2 (STromal Interaction Molecule 2)	APPswe/PSEN1dE9 mice	Learning and memory deficits↓, hippocampal neurogenesis↓, synaptic plasticity↓, Aβ deposition↑	Peng et al. (2024)
miRNA-128	↑	STIM2	AD mice	Synaptic function↓, memory imprecision↑	Deng et al. (2020)
miR-124	↓	BACE 1	APP/PS1 transgenic mice	autophagy↑	Du et al. (2017)

**Table 2:** Summary of miRNAs involved in apoptosis and inflammation in experimental animal models of Alzheimer diseases.

miRNAs	Expression	AD-relevant targets	Experimental animal models	Apoptosis	Inflammation	Ref.
miRNA-214–3p	↓	LC3βII and Beclin1	SAMP8 mice	↑	–	Zhang Y. et al. (2016)
miRNA-200a-3p	↑	SIRT1	APPswe/PS ΔE9 transgenic mice	↑	–	Zhang et al. (2017)
miRNA-155	↑	Tfeb	APP/PS1 mice, 5XFAD mouse	–	↑	Aloi et al. (2023)
miRNA-155	↑	SOCS-1	3xTg AD	–	↑	Guedes et al. (2014)
miRNA-155	↑	PI3K-Akt signaling	APPSWE/PS1L166P AD	–	↑	Shi et al. (2022)
miRNA-223-3p	↓	NLRP3/GSDMD	STZ-induced sAD	↑	↑	Lin Y. et al. (2024)
miRNA-137-5p	↓	USP30	Male C57BL/6 J	↑	–	Jiang et al. (2023)
miRNA-29c-3p	↓	BACE1	STZ-induced sAD	↑	–	Cao et al. (2021)
miRNA-142	↑	pCaMKII and BAI3	APP/PS1 transgenic mice	↑	–	Fu et al. (2021)
miRNA-146a	↓	FoxO6, Nkd2, Cd4 and Stpg1	APP/PS1 transgenic mice	↑	↑	Liang et al. (2021)
miRNA-223	↓	YB-1	APP/PS1 mice	–	↑	Wei et al. (2024)
miRNA-98-5p	↑	α7 nAChR	APP/PS1 mice	–	↑	Song C. et al. (2021)
miRNA-128	↑	PPARγ	3 × Tg-AD, 3 × Tg-AD-KO, WT C57BL/6	–	↑	Liu et al. (2019)

miRNA-132 plays a vital role in supporting neuronal health by promoting survival pathways and protecting against stress. It also helps regulate inflammatory responses in the nervous system, which is particularly important since chronic inflammation is a hallmark of AD. miRNA-132 is involved in mechanisms that improve learning and memory, highlighting its importance for cognitive functions. The study by Salta et al. (2016) consistently demonstrated that miRNA-132 levels are significantly decreased in AD, potentially contributing to the condition’s progression. The reduction of miRNA-132 worsens amyloid and tau pathology in models of AD. This indicates that miRNA-132 typically helps to alleviate these pathological processes. miRNA-132 regulates inositol 1,4,5-trisphosphate 3-kinase B (ITPKB). Without miRNA-132, ITPKB levels increase, which is associated with heightened activity of ERK1/2 and the APP-cleaving enzyme 1 (BACE1). Elevated ITPKB levels are also associated with increased phosphorylation of tau, resulting in tau aggregation, a key characteristic of AD. This research highlights the significance of miRNA-132 in slowing AD progression by regulating survival, inflammation, and memory processes. The downregulation of miRNA-132 results in pathological changes through ITPKB, impacting key pathways related to amyloid and tau pathology. This suggests potential targets for therapeutic intervention.

Tang et al. (2017) found that miRNA-139 levels were significantly higher in the hippocampus of aged senescence-accelerated mouse prone 8 (SAMP8) mice, a model used for studying aging and AD, compared to control mice. This finding suggests that miRNA-139 may play a role in age-related cognitive decline. Overexpression of miRNA-139 via hippocampal injection impaired hippocampus-dependent learning and memory processes. Conversely, reducing miRNA-139 levels resulted in improvements in cognitive functions, indicating that elevated miRNA-139 levels are detrimental to cognitive abilities. The study identified cannabinoid receptor type 2 (CB2) as a potential target gene of miRNA-139. An inverse relationship was observed between miRNA-139 expression and CB2 levels in primary hippocampal cells, indicating that higher miRNA-139 levels corresponded with lower CB2 levels. Moreover, miRNA-139 was found to negatively influence the response to pro-inflammatory stimuli, suggesting that elevated levels of miRNA-139 could exacerbate neuroinflammatory processes. This modulation may contribute to the neuroinflammation observed in AD. Overall, the findings suggest that miRNA-139 plays a pathogenic role in AD by regulating neuroinflammatory processes mediated by CB2, potentially contributing to the cognitive deficits associated with the disease.

miRNAs are recognized for their critical roles in neuronal development, survival, and apoptosis. Specifically, they can regulate important proteins associated with AD, such as BACE1, which is essential for the production of Aβ. Yang et al. (2015) examined the expression levels of the miRNA-29c family in peripheral blood samples from patients with AD and discovered that miRNA-29c levels were notably lower than those in age-matched controls. In contrast, BACE1 expression was significantly elevated in the blood of AD patients. Correlation analysis revealed an inverse relationship between miRNA-29c expression and BACE1 protein levels in the peripheral blood samples of these patients. This indicates that reduced levels of miRNA-29c could result in increased BACE1 levels, potentially facilitating the progression of AD. The researchers also explored the role of miRNA-29c in hippocampal neurons, conducting studies both *in vitro* and *in vivo*. They found that elevating miRNA-29c levels enhanced learning and memory behaviors in SAMP8 mice, a model that mimics accelerated aging. This improvement was linked to increased activity of protein kinase A (PKA) and cAMP response element-binding protein (CREB), both of which are involved in neuroprotection.

Recent research has identified that miRNAs, such as miRNA-200a-3p, are abnormally expressed in the brains of AD patients. Zhang et al. (2017) reported elevated levels of miRNA-200a-3p and reduced levels of SIRT1 in the hippocampus of APPswe/PS ΔE9 transgenic mice, which serve as a model for AD. Flow cytometry analysis revealed that exposure to Aβ 25–35 significantly increased the apoptosis rate and the expression of cleaved-caspase-3 in PC12 cells. Transfecting these cells with anti-miRNA-200a-3p resulted in a significant reduction in both the apoptosis rate and cleaved-caspase-3 activity. Additionally, an MTT assay indicated that the survival rate of PC12 cells exposed to Aβ 25–35 improved when treated with anti-miRNA-200a-3p compared to a control group. This suggests that inhibiting miRNA-200a-3p supports cell survival in the presence of Aβ. A dual-luciferase reporter gene assay confirmed that miRNA-200a-3p interacts with the predicted binding sites in the 3′ untranslated region (UTR) of SIRT1 mRNA, reinforcing its role as a regulatory miRNA. Moreover, downregulating SIRT1 exacerbated Aβ 25-35-induced neuronal apoptosis and increased cleaved-caspase-3 levels in PC12 cells. In contrast, treatment with anti-miRNA-200a-3p alleviated these effects, suggesting that the suppression of SIRT1 by miRNA-200a-3p contributes to neuronal apoptosis.

Many research efforts have focused on the aggregation of Aβ, particularly Aβ42, which seems to play a crucial role in the onset of AD. In individuals with AD, Aβ42 is present in high concentrations and initiates the polymerization process, resulting in the creation of neurotoxic lamellar structures (Benilova et al., 2012).

miRNA-155 is especially recognized for its involvement in inflammation regulation and has been associated with several neurodegenerative diseases, including AD. Aloi et al. (2021) suggested that the expression of miRNA-155 in microglia influences their capacity to internalize and degrade extracellular fibrillar Aβ1-42 (fAβ1-42). After stimulation with lipopolysaccharide, primary microglia showed a quick increase in miRNA-155 expression, followed by a later rise in miRNA-146a, which is known for its anti-inflammatory properties. This suggests that miRNA-155 plays a role in triggering inflammatory responses. Conditional overexpression of miRNA-155 in microglia led to a significant rise in miRNA-146a levels, indicating a regulatory interaction between these two miRNAs. In contrast, the conditional removal of miRNA-155 facilitated the transport of fAβ1-42 to low-pH compartments, where degradation generally occurs, suggesting that miRNA-155 plays a negative role in the breakdown of fAβ1-42. Changes in miRNA-155 expression—whether increased or decreased—resulted in an increased uptake of fAβ1-42 through the plasma membrane, emphasizing the complex interplay between miRNA-155 and Aβ internalization. In another study, Aloi et al. (2023) found that miRNA-155 plays a dual role in AD pathology. It influences microglial responses to Aβ, driving inflammation and promoting Aβ accumulation. In contrast, deleting miRNA-155 shifts microglial activity toward a more anti-inflammatory state, which increases Aβ clearance; however, this change may also result in hyperexcitability and related seizure issues. miRNA-155 is recognized as a key transcriptional regulator in the TREM2-APOE pathway, influencing the characteristics of MGnD microglia. This suggests that miRNA-155 is significant in determining how microglia react to amyloid pathology. Single-cell RNA sequencing has identified new microglial subtypes referred to as neurodegeneration-associated microglia (MGnD) or disease-associated microglia (DAM), which become activated in the vicinity of cerebral amyloid plaques in AD.

In the research conducted by Guedes et al. (2014), a significant increase in miR-155 levels was observed in the brains of 12-month-old 3xTg AD mice. This elevation in miR-155 occurred alongside heightened activation of microglia and astrocytes, and it was noted before the formation of extracellular Aβ aggregates. This finding implies that earlier Aβ forms, such as oligomers, could trigger initial neuroinflammatory responses. The study also examined the relationship between miR-155 and the c-Jun transcription factor in the activation of glial cells by Aβ. Results showed that both miR-155 and c-Jun are upregulated early in the disease, leading to the production of inflammatory mediators like IL-6 and IFN-β. This inflammatory response is associated with a reduction in SOCS-1 levels, which depends on miR-155. Additionally, silencing c-Jun resulted in decreased levels of miR-155 in Aβ-activated glial cells, indicating that targeting miR-155 could be a promising approach for controlling neuroinflammation in AD.

Despite the increasing interest in investigating AD manifestations in the retina—a CNS organ amenable to non-invasive imaging—MGnD microglia had not been examined in this context before. Shi et al. (2022) found elevated levels of Clec7a+ and Galectin-3+ MGnD microglia in the retinas of transgenic APPSWE/PS1L166P AD model mice, suggesting a notable activation of these microglial subtypes in response to amyloid pathology. By selectively targeting MGnD microglia through the ablation of miRNA-155 using a tamoxifen-inducible CreERT2 system in APPSWE/PS1L166P mice, the researchers observed a reduction in retinal Clec7a+ and Galectin-3+ microglial populations, accompanied by an increase in homeostatic P2ry12+ microglia. This indicates that miRNA-155 plays a role in sustaining the dysfunctional state of activated microglia. The depletion of MGnD microglia was associated with the preservation of the inner blood-retina barrier and a decrease in vascular amyloidosis, underscoring the potential protective benefits of targeting these microglial populations for retinal health. Additionally, reducing miRNA-155 in microglia resulted in lower retinal inflammation, highlighting miRNA-155’s role in regulating inflammatory responses in the retina during AD. Mass spectrometry analysis revealed increased PI3K-Akt signaling in the retinas of mice deficient in microglial miRNA-155. Furthermore, predicted reductions in interleukin-8 (IL-8) and secreted phosphoprotein 1 (Spp1) levels were observed, indicating that miRNA-155 is involved in the regulation of inflammatory cytokine production.

On the other hand, miRNA-155 is essential for the effective functioning of immune cells, such as T and B lymphocytes. It controls genes related to inflammation and immune signaling, positioning it as a critical component in both innate and adaptive immune responses. In the brain, miRNA-155 plays a role in regulating neuroinflammatory responses. Its expression can be stimulated by different factors, including infections and injuries, which activate inflammatory pathways. Readhead et al. (2018) demonstrated that miRNA-155 might affect the buildup of Aβ plaques, a defining feature of AD. For example, the removal of miRNA-155 has been linked to greater Aβ accumulation in mouse models. Additionally, miRNA-155 is influenced by viral infections, such as those caused by human herpesvirus-6A (HHV-6A), which can reduce its expression. This interaction could connect viral activity to the development of neurodegenerative diseases.

miRNA-146a is a small RNA molecule consisting of 22 nucleotides and is significantly overexpressed in the brains of individuals with AD. This increased expression indicates a possible role in the inflammatory processes associated with the disease. miRNA-146a targets multiple mRNAs involved in inflammation and membrane functions. Key targets of miRNA-146a include Complement Factor H (CFH), which is crucial for regulating the complement system and contributes to immune responses, and Interleukin-1 Receptor Associated Kinase-1 (IRAK-1), an important mediator in inflammatory signaling pathways. Significant reductions in the expression levels of these targets were observed when miRNA-146a was upregulated. The researchers assessed the levels of miRNA-146a, CFH, IRAK-1, and Tetraspanin-12 (TSPAN12) in different cell types, including primary human neuronal-glial (HNG) co-cultures, human astroglial (HAG), and microglial (HMG) cells, which were subjected to stress from Aβ42 peptide and TNF-α. The findings demonstrated a consistent inverse relationship between miRNA-146a levels and the expression of CFH, IRAK-1, and TSPAN12 across various cell types. This indicates that increased levels of miRNA-146a are associated with decreased levels of these inflammatory mediators. HAG cells displayed the strongest interaction between miRNA-146a and IRAK-1, whereas HNG cells showed the most pronounced response concerning miRNA-146a and TSPAN12. HMG cells, which act as the brain’s resident scavenging macrophages, demonstrated the most notable changes related to miRNA-146a and CFH. By downregulating pro-inflammatory mediators, miRNA-146a could contribute to the reduction of neuroinflammation, which is a key feature of AD.

miRNA-132 is observed to be downregulated in the brains of individuals with AD, suggesting its potential importance in disease progression and cognitive decline. This miRNA is involved in regulating synaptic plasticity, which is vital for learning and memory, by affecting the expression of genes essential for synaptic function and stability, thereby enhancing neuronal communication. The research conducted by Walgrave et al. (2021) found that miRNA-132 targets multiple mRNAs linked to inflammation and neurodegeneration. By modulating these targets, miRNA-132 may help reduce the neuroinflammatory responses that are frequently heightened in AD. It works by suppressing the expression of several pro-inflammatory cytokines and signaling pathways associated with neuroinflammation, thereby shielding neurons from inflammatory damage, which is a crucial factor in AD pathology. Furthermore, the study indicates that miRNA-132 may affect the processing of APP and the production of Aβ. By regulating these pathways, miRNA-132 might help decrease the formation of amyloid plaques, which are a hallmark of AD. A reduction in miRNA-132 levels is associated with cognitive impairments observed in AD models, whereas restoring its levels has been shown to improve cognitive function. This suggests that boosting miRNA-132 expression could serve as a promising therapeutic strategy to alleviate memory deficits. Due to its protective functions in maintaining synaptic integrity and combating neuroinflammation, miRNA-132 appears to be a promising target for therapeutic strategies. Methods designed to increase miRNA-132 levels or replicate its effects could be advantageous in treating AD by restoring synaptic health and minimizing inflammatory damage. In relation to AD, miRNA-98-5p has been demonstrated to inhibit the expression of the α7 nicotinic acetylcholine receptor (nAChR), a receptor vital for cognitive function and the regulation of neuroinflammation. By modulating the levels of α7 nAChR, miRNA-98-5p affects the cognitive deficits seen in AD models. Reduced levels of miRNA-98-5p can lead to increased expression of α7 nAChR, resulting in better cognitive outcomes. The downregulation of miRNA-98-5p results in the inhibition of the Nuclear Factor kappa B (NF-κB) signaling pathway, which plays a key role in inflammatory responses. This modulation aids in reducing neuroinflammation related to AD. The reduction of miRNA-98-5p activates calcium (Ca^2+^) signaling pathways, which enhances neuronal function and decreases Aβ accumulation, a characteristic of AD. The interaction between miRNA-98-5p and α7 nAChR represents a promising therapeutic target. Approaches designed to adjust miRNA-98-5p levels could aid in mitigating cognitive decline and neuroinflammation associated with AD.

The research conducted by Badr et al. (2024) highlighted that microglia in the brains of AD patients exhibit elevated levels of miRNA-17. This increase is linked to disrupted autophagy, Aβ accumulation, and heightened production of inflammatory cytokines, suggesting that miRNA-17 may contribute to the progression of AD pathology. To selectively inhibit miRNA-17 in microglia, the researchers developed mannose-coated lipid nanoparticles (MLNPs) that carry an antagomir targeting miRNA-17 (Anti-17 MLNPs). These nanoparticles are engineered to attach to mannose receptors, which are abundantly expressed on microglia, enabling precise delivery. The research employed the 5XFAD mouse model, which displays various characteristics of AD. The injection of Anti-17 MLNPs into the intra-cisterna magna allowed the researchers to successfully deliver the antagomir to microglia, leading to a significant reduction of miRNA-17 in these cells, while not affecting neurons, astrocytes, or oligodendrocytes. Treatment with Anti-17 MLNPs led to reduced inflammation, improved autophagy, and lower Aβ levels in the brains of 5XFAD mice. This indicates that targeting miRNA-17 may help mitigate various pathological features associated with AD. Additionally, the treatment enhanced spatial memory and reduced anxiety-like behaviors in the AD mouse model, suggesting that inhibiting miRNA-17 not only targets pathological problems but also improves cognitive function.

Patients with AD exhibited lower levels of miRNA-451a in their cerebrospinal fluid (CSF), which showed a positive correlation with cognitive assessment scores and a negative correlation with depression scales. This suggests that decreased miRNA-451a levels are associated with impaired cognitive function and increased depressive symptoms. In the medial prefrontal cortex (mPFC) of APP/PS1 transgenic mice, miRNA-451a levels were notably diminished in both neurons and microglia, highlighting that miRNA-451a is negatively influenced by AD pathology. To explore the functional role of miRNA-451a, Feng et al. (2023) injected AAV9-miRNA-451a-GFP into the mPFC of APP/PS1 mice. Four weeks post-injection, various behavioral and pathological evaluations indicated that elevated levels of miRNA-451a enhanced behavioral deficits and pathological characteristics linked to AD, including long-term memory problems, depression-like behaviors, β-amyloid accumulation, and neuroinflammation. Mechanistically, miRNA-451a was shown to reduce the expression of BACE1 in neurons, an essential enzyme involved in Aβ production. This effect occurs through the inhibition of the Toll-like receptor 4 (TLR4)/IκB Kinase β (IKKβ)/NF-κB signaling pathway. Additionally, miRNA-451a also decreased microglial activation by inhibiting the NOD-like receptor protein 3 (NLRP3), which is involved in inflammatory responses.

MSC-derived small extracellular vesicles (sEVs) have shown the capacity to modulate inflammatory responses, positioning them as a promising therapeutic option for AD. Induced pluripotent stem cell (iPSC)-derived MSCs offer particular benefits due to their low immunogenicity and reduced variability. Lin L. et al. (2024) used a streptozotocin (STZ)-induced sporadic mouse model of AD (sAD) to assess the anti-inflammatory effects of iPSC-MSC-sEVs. The intracisternal administration of these vesicles resulted in decreased neuroinflammation, reduced amyloid deposition, and lower neuronal apoptosis, thereby improving cognitive dysfunction. The researchers examined the role of miRNA-223-3p in mediating the anti-inflammatory effects of MSC-derived sEVs and found that miRNA-223-3p directly targets NLRP3, a crucial component of the inflammasome that contributes to neuroinflammation. Inhibiting miRNA-223-3p almost entirely negated the reduction of NLRP3 achieved by MSC-sEVs. This discovery underscores miRNA-223-3p as a vital mediator of the anti-inflammatory effects of MSC-derived sEVs, emphasizing its important role in modulating the inflammatory response linked to AD. The study’s findings indicate that intracisternal delivery of iPSC-MSC-sEVs can effectively reduce cognitive deficits in the sAD mouse model by inhibiting the NLRP3/GSDMD-mediated neuroinflammatory pathway.

miRNA-223 is recognized for its role in regulating inflammatory responses, especially within immune cells. Within the CNS, miRNA-223 has been demonstrated to safeguard neurons by decreasing neuroinflammation and enhancing neuronal survival (Wei et al., 2024). It plays a crucial role in balancing inflammation and repair processes. miRNA-223 is implicated in the sorting and release of exosomes from cells, especially in microglia. These exosomes can transport miRNA-223 and other signaling molecules to affect neighboring cells and adjust the microenvironment. In AD models, miRNA-223 has been associated with the activation of microglia (Wei et al., 2024). Activated microglia release exosomes rich in miRNA-223, aiding in reducing neuroinflammation and neuronal damage. miRNA-223 also interacts with Y-box binding protein 1 (YB-1), which assists in incorporating miRNA-223 into microglial exosomes (Wei et al., 2024). This interaction is essential for the neuroprotective effects of miRNA-223 in AD. Due to its ability to reduce neuroinflammation and support neuroprotection, miRNA-223 is a promising target for therapeutic strategies in AD. Boosting the release of miRNA-223-enriched exosomes from microglia may enhance neuronal health and cognitive function.

Levels of miRNA-29a rise with age in both humans and mice. In the brain, it is involved in neuronal maturation and the response to inflammatory signals. Research has shown that elevated levels of miRNA-29a in the human brain are associated with faster cognitive decline before death. This implies that lowering miRNA-29a levels might help improve memory deficits related to AD. To evaluate this hypothesis, Mei et al. (2024) engineered an adeno-associated virus (AAV) that expressed either a green fluorescent protein (GFP) with a miRNA-29a “sponge” or a control empty vector. The purpose of the miRNA-29a sponge was to effectively reduce miRNA-29a levels. Upon administering the AAV containing the miRNA-29a sponge to the hippocampi of both 5 × FAD AD model mice and wild-type (WT) mice, they observed a significant decrease in miRNA-29a levels. Administering the AAV that expressed the miRNA-29a sponge to the hippocampi of both 5 × FAD AD model mice and wild-type (WT) mice caused a notable decrease in miRNA-29a levels. This intervention also resulted in enhanced performance in memory assessments, particularly in the Morris water maze and fear conditioning tests. The miRNA-29a sponge substantially reduced beta-amyloid accumulation in the hippocampus of 5 × FAD mice, which is a key feature of AD pathology. This decrease suggests a possible protective effect against amyloid-related toxicity. Additionally, the treatment lowered the activation of astrocytes and microglia in both 5 × FAD and wild-type (WT) mice, indicating that reducing miRNA-29a might help alleviate neuroinflammatory responses linked to AD. Using transcriptomic and proteomic sequencing, the researchers discovered two potential effectors, Plxna1 and Wdfy1, at the transcript and protein levels in wild-type (WT) and 5 × FAD mice, respectively. These targets may play a role in the mechanisms through which miRNA-29a affects cognitive function and neuroinflammation.

miRNA-137-5p protects the development and progression of AD by mitigating Aβ1-42 neurotoxicity and supporting neuronal health. Lower levels of miRNA-137-5p have been observed in the brains of individuals with AD. In the study conducted by Jiang et al. (2023) bioinformatics analysis and dual-luciferase reporter assays were used to explore the interaction between miRNA-137-5p and USP30, a protein involved in deubiquitination that may contribute to neurodegenerative processes. In SH-SY5Y neuroblastoma cells, miRNA-137-5p mimics effectively mitigated neurotoxicity caused by Aβ1-42. However, this protective effect was diminished when USP30 was overexpressed, suggesting that USP30 may counteract the positive effects of miRNA-137-5p. In mouse models of AD, administering miRNA-137-5p led to significant improvements in cognitive function and mobility. Significantly, it decreased Aβ1-42 deposition, Tau hyperphosphorylation, and neuronal apoptosis in the hippocampus and cortex, which are crucial regions impacted by AD. The study demonstrated that miRNA-137-5p effectively lowered USP30 levels in the brains of AD mice. In contrast, the overexpression of USP30 partially reduced the benefits in AD symptoms brought about by miRNA-137-5p, indicating that downregulating USP30 is a vital mechanism through which miRNA-137-5p exerts its effects.

The study by Garcia et al. (2021) explored the intricate role miR-124 in modulating neuronal dynamics and inflammatory responses in SH-SY5Y APPSwe and PSEN1 Mutant iPSC-Derived AD Models. The findings indicated that miR-124 regulates several critical genes associated with AD pathology, one of the most notable being BACE1 (beta-secretase 1). BACE1 is essential for the cleavage of APP, a process that produces Aβ peptides, which accumulate to form plaques—a hallmark of AD (An et al., 2017). By targeting and downregulating BACE1 expression, miR-124 can help reduce Aβ production, potentially alleviating one of the primary contributors to the neurodegenerative processes in AD. Furthermore, the study highlighted the importance of the miR-124/PTPN1 signaling pathway (Garcia et al., 2021). Abnormalities in this pathway exacerbate tau pathology, another critical aspect of AD characterized by the hyperphosphorylation and aggregation of tau protein (Garcia et al., 2021; Hou et al., 2020). Correcting these signaling abnormalities through miR-124 modulation resulted in the rescue of tau-related dysfunctions. This finding underscores the potential of miR-124 as a therapeutic target, suggesting that enhancing its expression or mimicking its function could provide a novel strategy for addressing tau pathology in AD.

XPO1 is responsible for transporting proteins and RNA from the nucleus to the cytoplasm. It identifies and binds to cargo proteins that have a nuclear export signal (NES). In the context of AD, research has indicated that increased levels of XPO1 can worsen neurodegeneration by promoting the export of protective factors and the buildup of harmful proteins. MiRNAs, such as miRNA-483-3p, can target XPO1 to regulate its expression (Luo et al., 2022). For instance, in studies like that of Luo et al., the downregulation of miRNA-483-3p leads to higher levels of XPO1, which is associated with increased apoptosis and cognitive deficits.

In the research conducted by Cao et al. (2021) the roles and molecular mechanisms of miRNA-29c-3p in AD are examined, with a particular emphasis on its impact on cell proliferation, apoptosis, and the regulation of β-site BACE1. To create AD animal models, STZ was injected into the lateral ventricle of mice, while cell models were induced using 10 μM Aβ. These models were utilized to investigate the effects of miRNA-29c-3p on processes related to AD. The study revealed that miRNA-29c-3p levels were significantly lower, while BACE1 levels were higher in both the brain tissues of AD animal models and in Aβ-treated cell models. Cells exposed to Aβ showed a notable decrease in proliferation and an increase in apoptosis, along with heightened phosphorylation of tau protein. However, overexpressing miRNA-29c-3p or silencing BACE1 resulted in increased cell proliferation and decreased apoptosis by altering the levels of related proteins. In contrast, inhibiting miRNA-29c-3p or overexpressing BACE1 exacerbated the negative effects caused by Aβ, highlighting the importance of both factors in mediating cellular responses in AD. Rescue experiments confirmed that BACE1 is a functional target of miRNA-29c-3p. The negative regulation of BACE1 by miRNA-29c-3p was found to significantly impact the progression of AD.

miRNA-142-5p has been linked to various neurodegenerative diseases, yet its specific function in AD remains unclear. Fu et al. (2021) showed that miRNA-142-5p significantly contributes to the development and progression of AD by regulating BAI3 expression and affecting neuronal responses to Aβ toxicity. BAI3, a member of the adhesion-G protein-coupled receptor subgroup, is mainly expressed in neurons and is thought to play a role in neuropsychiatric disorders. The researchers found altered levels of miRNA-142-5p in the hippocampus of AD mice, suggesting its potential role in the pathology of AD. Inhibiting miRNA-142-5p resulted in increased BAI3 expression, which correlated with enhanced neuronal viability and decreased apoptosis in Aβ-exposed neurons. Additionally, the levels of phosphorylated Synapsin I and calcium/calmodulin-dependent protein kinase II (CaMKII), along with the expression of postsynaptic density protein 95 (PSD-95), were significantly restored in the hippocampus of APP/PS1 transgenic mice after inhibiting miRNA-142-5p. In cultured neurons, inhibiting miRNA-142-5p was found to decrease the levels of Aβ1-42, β-APP, BACE-1, and presenilin-1 (PS-1), all of which are key components in the pathology of AD. These findings pointed to BAI3 as a possible target gene for miRNA-142-5p, suggesting that miRNA-142-5p negatively regulates BAI3, which subsequently affects neuronal health and synaptic function. A major pathological feature of AD is the abnormal polarization of microglia. Although microglia can protect neurons by clearing Aβ and tau, they frequently shift to a pro-inflammatory phenotype in the context of AD. This change reduces their phagocytic activity, damages neurons, and worsens the pathology of AD. Liang et al. (2021) reported a link between a polymorphism in miRNA-146a and the risk of sporadic AD. They also discovered that nasal delivery of miRNA-146a mimics enhanced cognitive function and diminished key pathological traits of AD. Additionally, overexpressing miRNA-146a specifically in microglia resulted in fewer cognitive deficits associated with learning and memory in APP/PS1 transgenic mice. Overexpression of miRNA-146a reduced neuroinflammation, lowered Aβ levels, and improved neuritic pathology associated with plaques, while also preventing neuronal loss. Liang et al. discovered that miRNA-146a could shift the microglial phenotype from a pro-inflammatory state to a more neuroprotective one. This transition involved a decrease in pro-inflammatory cytokines and an increase in phagocytic activity, thus safeguarding neurons both *in vitro* and *in vivo*. Researchers discovered that miRNA-146a could transform the microglial phenotype from a pro-inflammatory state to a more neuroprotective one. This transformation included a decrease in pro-inflammatory cytokines and an increase in phagocytic activity, effectively protecting neurons both in vitro and in vivo. Additional analysis revealed that miRNA-146a countered AD pathology mainly through mechanisms associated with neuroinflammation, highlighting its role in regulating inflammatory responses in the brain.

Studies have revealed that Gram-negative bacterial lipopolysaccharides (LPS) are present in neurons impacted by AD, particularly in the neocortex and hippocampus. LPS functions as a pro-inflammatory agent, activating inflammatory pathways, including those mediated by NF-kB. Pogue et al. (2022) discovered that miRNA-30b-5p is a microRNA that is increased in the brains of AD patients and in human neuronal-glial (HNG) cells exposed to LPS. This miRNA responds to NF-kB signaling, which is triggered by inflammatory stimuli. miRNA-30b-5p targets the 3′-untranslated region (3’-UTR) of neurofilament light chain (NF-L) mRNA, resulting in a post-transcriptional decrease in NF-L expression. NF-L is crucial for maintaining the structural integrity of neurons, as it is involved in the cytoskeleton and synaptic organization. A deficiency in NF-L is associated with neuronal atrophy and synaptic disturbances, which contribute to the neurodegenerative changes observed in AD. The study further highlighted that miRNA-30b-5p is significantly elevated in animal and cell models that have been exposed to Aβ peptides. Aβ can enhance the entry of LPS into neurons, connecting these pathological processes. The increased levels of miRNA-30b-5p contribute to neuronal damage, loss, inflammation, and impaired synaptic transmission, worsening neurodegeneration. Pogue et al. (2022) describe a pathological signaling network involving LPS, NF-kB, miRNA-30b-5p, and NF-L, illustrating a connection between LPS from gut microbiota and the inflammatory changes that result in cytoskeletal disorganization and impaired synaptic signaling in AD.

miRNA-132-3p plays a role in regulating the inflammatory response in the brain. It aids in controlling the activation of microglia, the brain’s native immune cells, thereby impacting neuroinflammation, a key factor in neurodegenerative diseases. Studies have demonstrated that miRNA-132-3p levels are significantly lower in the brains and blood of AD and APP/PS1 mice (Fu et al., 2022). This reduction is associated with cognitive decline and the progression of AD-related pathology. miRNA-132-3p targets PPM1F (protein phosphatase, Mg^2+^/Mn^2+^ dependent 1F), which plays a crucial role in regulating various signaling pathways related to neuronal health and function (Fu et al., 2022). It controls the expression of FOXO3a, a transcription factor that plays a role in stress resistance and neuronal survival (Fu et al., 2022). When miRNA-132-3p is dysregulated, it results in neuronal damage, inflammation, and synaptic failure, which are all critical to the advancement of AD. By influencing key proteins in these pathways, miRNA-132-3p impacts the onset and development of cognitive impairments (Fu et al., 2022).

Hu Y. B. et al. (2021) discovered an amyloid plaque-associated microenvironment (APAM) characterized by the spatial co-occurrence of AD-related pathophysiology, such as neuronal cell death, inflammatory signaling, and endolysosomal dysfunction surrounding amyloid plaques. Abnormal levels of miRNA-425 are associated with this microenvironment, underscoring its important role in the neuronal changes seen in AD. miRNA-425 is a neuron-specific microRNA that is reduced in the brains of people with AD. This decrease disrupts the normal spatial transcriptome of neurons, contributing to the complexity and variability of AD pathology. In a mouse model lacking miRNA-425, researchers noted increased APP processing, heightened neuroinflammation, greater neuronal loss, and cognitive impairments, suggesting that the absence of miRNA-425 worsens the pathological features of AD. On the other hand, in APP/PS1 mouse models, restoring miRNA-425 levels improved APAM-related changes and memory deficits, highlighting its potential as a therapeutic target. The study revealed a new mechanism by which the dysregulation of miRNA-425 affects spatial transcriptomic changes in the brains of individuals with AD. By maintaining a normal spatial transcriptome, miRNA-425 may help mitigate amyloid pathogenesis and related neurodegenerative processes.

Research has demonstrated that miRNA-27a levels are modified in the brains of AD patients and models, typically exhibiting increased expression in response to Aβ toxicity (Hu Y. et al., 2021). miRNA-27a negatively regulates SOX8, a transcription factor that provides protective effects for neurons (Hu Y. et al., 2021). By inhibiting SOX8, miRNA-27a may increase neuronal vulnerability and lead to apoptosis. Activation of SOX8 can lead to increased levels of β-catenin, which are vital for cell survival. Consequently, the inhibition of SOX8 by miRNA-27a may indirectly promote apoptosis and neuroinflammation. Modifying miRNA-27a levels, as shown in studies using compounds like notoginsenoside R2, can enhance neuronal survival and reduce inflammation, suggesting possible therapeutic advantages for AD (Hu Y. et al., 2021). Research indicates that targeting miRNA-27a could improve cognitive functions in AD models, highlighting its role in the cognitive decline associated with the disease.

miRNA-96-5p is involved in how cells respond to oxidative stress and inflammation, affecting pathways that protect against cellular damage. It is expressed in the brain and is vital for neuronal function, influencing processes like neuronal survival, differentiation, and communication. In AD, miRNA-96-5p is believed to exert a potentially inhibitory effect, with changes in its expression levels possibly contributing to the disease’s progression. It regulates Forkhead box protein O1 (Foxo1), a transcription factor that plays a role in stress responses and metabolism (Wu et al., 2020). By targeting Foxo1, miRNA-96-5p can influence various downstream effects related to oxidative stress and inflammation. Wu et al. (2020) showed that lowering levels of miRNA-96-5p can result in higher levels of antioxidant enzymes such as superoxide dismutase and catalase, which help reduce oxidative damage in neurons. Furthermore, miRNA-96-5p influences the expression of lipocalin-2 (Lcn2), a protein associated with inflammation and neurodegeneration. By regulating Foxo1 to inhibit Lcn2, it may be possible to mitigate the neuroinflammatory processes related to AD. Due to its role in important pathways associated with oxidative stress and inflammation, miRNA-96-5p could serve as a potential therapeutic target for AD. Modifying levels of miRNA-96-5p may influence the progression of the disease and improve neuronal health.

miRNA-130a-3p is involved in pathways that control cell survival and apoptosis, particularly in stressful conditions. By regulating both pro-apoptotic and anti-apoptotic factors, it helps ensure a balance in cell fate decisions. Its expression in neuronal tissues is vital for neuronal health and cognitive function, affecting processes like synaptic plasticity and neuroprotection. In AD models, levels of miRNA-130 a-3p are frequently reduced, which may contribute to neurodegeneration and cognitive decline. Wang et al. (2021) found that increasing the expression of miRNA-130a-3p can decrease apoptosis induced by Aβ, a key pathological feature of AD, suggesting its protective role against neuronal cell death. Moreover, miRNA-130a-3p targets Death-Associated Protein Kinase 1 (DAPK1), a protein that plays a role in apoptosis and stress responses. By regulating DAPK1 levels, miRNA-130a-3p can influence the neurotoxic effects linked to Aβ accumulation. In APP/PS1 mice, elevated levels of miRNA-130a-3p correlate with enhanced cognitive function, as indicated by behavioral tests like the Morris water maze, which reflect improved learning and memory.

## Polyphenols: natural compounds with therapeutic benefits

Polyphenols are organic compounds defined by having multiple phenolic groups in their structure (Zagoskina et al., 2023). Mainly sourced from plants, these compounds are present in seeds, stems, and almost all other parts of the plant (Zagoskina et al., 2023). They can also be produced as semi-synthetic and synthetic forms. Polyphenols are classified into various subgroups, including hydroxybenzoic acids, hydroxycinnamic acids, flavonoids, stilbenes, and lignans (Lang et al., 2024). Among these, flavonoids represent the largest and potentially most important group, encompassing flavonols, flavones, isoflavones, flavanones, anthocyanidins, and flavanols (Lang et al., 2024). The structural features and number of phenolic groups in polyphenols affect their various chemical properties, allowing them to scavenge oxygen radicals and interact with other molecules in food (Lang et al., 2024). Given their prevalence in natural foods, polyphenols have been utilized in traditional medicine and are currently being studied for their potential benefits in a range of health issues, including neurodegenerative diseases (Li Z. et al., 2023). They are involved in both *in vitro* and *in vivo* studies, with a growing number of clinical trials aimed at evaluating their effects (Li Z. et al., 2023). Polyphenols exert their influence through various mechanisms, including the modulation of key signaling pathways that affect a range of cellular functions, such as AKT/PI3K signaling, NF-κB signaling, ERK signaling, MAPK signaling, Nrf2 signaling, AMPK signaling, BDNF/CREB signaling, Wnt/β-Catenin signaling, PI3K/Akt signaling, JAK/STAT signaling, and several pathways associated with growth factors (Vauzour, 2012; Yan et al., 2022; Tayab et al., 2022; Long et al., 2021).

## Beneficial effects of polyphenols in neurological diseases

Neurodegenerative diseases are strongly linked to the aging process and primarily impact older adults. The ongoing generation of reactive oxygen species (ROS), increased inflammatory responses, and the resulting activation of apoptosis or autophagy render neurons susceptible to functional decline, as evident in AD. A review by Syarifah-Noratiqah et al. (2018) has highlighted three polyphenolic compounds—Epigallocatechin-3-gallate, Resveratrol, and Curcumin —as potential candidates for treating AD. Colizzi (2019) reached similar conclusions; however, more evidence is required to validate polyphenols as standard treatment options. It is believed that polyphenols affect AD by decreasing amyloidogenesis and inflammation, thus safeguarding neuronal cells that already have amyloid deposits. Furthermore, polyphenols block the activation of signaling pathways linked to amyloid deposition, including the NF-κB/IL-1β/NLRP3 signaling cascade.

## Interaction between polyphenols and miRNA regulation in Alzheimer disease

**Figure 4:**
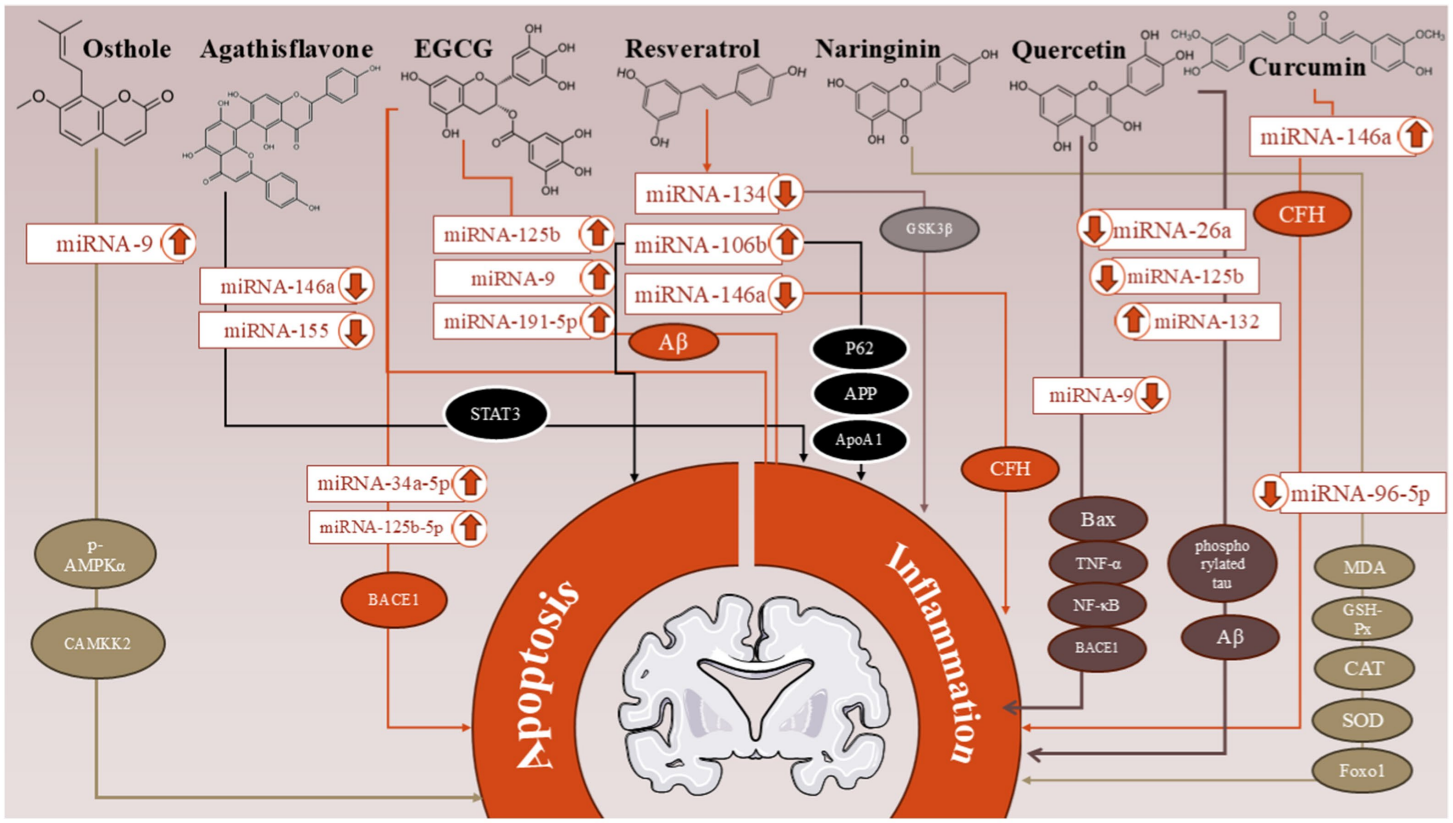
The positive effects of various polyphenols on miRNAs in Alzheimer’s disease (AD) are summarized in this figure, highlighting the roles of Resveratrol, Quercetin, Curcumin, Epigallocatechin-3-Gallate (EGCG), Naringenin, Agathisflavone, and Osthole. These compounds demonstrate their potential in preventing inflammation and apoptosis through specific molecular pathways.

The possible neuroprotective effects of polyphenols in AD have attracted much attention, primarily because of their functions in lowering inflammation and apoptosis caused by dysregulated miRNAs. Specific miRNAs are dysregulated in AD, which leads to inflammation and apoptosis. (Table 2), which are pathological features of the illness. Significant dysregulated miRNAs include miR-146a, which is implicated in inflammatory responses (Gong and Sun, 2022); miR-132, which is linked to tau disease and neuronal death (Mu et al., 2024); and miR-155, which is related to increased neuroinflammation (Aloi et al., 2021). Potent polyphenols and antioxidants protect neurons from damage by reducing oxidative stress, which can cause apoptosis. In addition, inflammatory pathways can be changed by lowering the expression of pro-inflammatory cytokines and promoting a stronger neuroinflammatory response. The positive effects of various polyphenols on miRNAs in AD are summarized in Table 3 and Figure 4, highlighting their roles in preventing inflammation and apoptosis through specific pathways.

**Table 3:** Role polyphenols in reducing apoptosis and inflammation by dysregulated miRNAs in Alzheimer diseases (AD).

Polyphenol	miRNAs	Up/down expression	Target	Apoptosis	Inflammation	Ref.
Resveratrol-selenium	miRNA-134	↓	GSK3β	–	↓	Abozaid et al. (2022)
Resveratrol	miRNA-106b	↑	APP, P62, and ApoA1	↓	↓	Kong et al. (2016)
Resveratrol analog CAY10512	miRNA-146a	↓	CFH	–	↓	Lukiw et al. (2008)
Curcumin	miRNA-146a	↑	CFH	–	↓	Gong and Sun (2022)
Quercetin	miRNA-9	↓	TNF-α, NF-κB, AβPP, BACE1, Bax	↓	↓	Ebrahimpour et al. (2020)
Quercetin	miRNA-132, miRNA-125b, miRNA-26a	↑ ↓ ↓	Aβ, phosphorylated tau	–	↓	Lv et al. (2018)
Epigallocatechin-3-Gallate (EGCG)	miRNA-125b-5p, miRNA-34a-5p	↑ ↑	BACE1	↓	↓	Li et al. (2020)
Epigallocatechin-3-Gallate (EGCG)	miRNA-191-5p, miRNA-9, miRNA-125b	↑ ↑ ↑	Aβ	–	↓	Hong et al. (2017)
Agathisflavone	miRNA-155, miRNA-146a	↓ ↓	STAT3	–	↓	Dos Santos et al. (2024)
Osthole	miRNA-9	↑	CAMKK2 and phosphorylated AMPKα (p-AMPKα)	↓	–	Li et al. (2016)
Naringin	miRNA-96-5p	↓	Foxo1, Lcn2, SOD, CAT, and GSH-Px, hydroxyproline (OH-), MDA	–	↓	Wu et al. (2020)

## Resveratrol

Resveratrol, also known as 3,5,4′-trihydroxytrans-stilbene, is an edible naturally occurring phytoalexin. Red wine, peanuts, grapes, and several other foods contain it. It has remarkable therapeutic potential. Resveratrol has been shown to play a part in avoiding several age-related illnesses, including cancer, neurological disorders, and cardiovascular conditions (Nowacka et al., 2024; Zhou et al., 2021). Additionally, resveratrol contains antioxidant and anti-inflammatory qualities (Santos et al., 2023). It has a well-established pathophysiological role in neurodegenerative diseases like AD (Zhou et al., 2021). Numerous studies have shown that resveratrol can control the expression of miRNAs, which are unregulated in AD.

## miRNA-134

Resveratrol is recognized for its neuroprotective properties, especially in relation to neurodegenerative diseases such as AD. However, its low bioavailability and solubility limit its effectiveness in living organisms. The research conducted by Abozaid et al. (2022) presented RSV-SeNPs, which improve the delivery and effectiveness of resveratrol. Selenium is a vital micronutrient that contributes to brain health and may enhance the effects of resveratrol. RSV-SeNPs were shown to reduce oxidative markers and mitochondrial dysfunction, both important factors in the development of AD. Administering RSV-SeNPs resulted in decreased levels of Aβ, which are linked to neurotoxicity and cognitive deterioration in AD. This reduction is crucial for restoring cholinergic function, which is frequently impaired in AD. The research by Abozaid et al. (2022) emphasized the activation of the phosphatidylinositol 3-kinase (PI3K) pathway, which subsequently inactivates GSK-3β. This is important because GSK-3β plays a role in the hyperphosphorylation of tau protein, a key feature of AD. Hyperphosphorylated tau aggregates into neurofibrillary tangles. These tangles are toxic to neurons and are linked to cognitive decline and other symptoms of AD. By inhibiting GSK-3β, RSV-SeNPs may help prevent neurodegeneration associated with tau. Additionally, RSV-SeNPs were found to downregulate the expression of signal transducer and activator of transcription (STAT3) and interleukin-1β (IL-1β), both of which are indicators of neuroinflammation. This decrease in inflammation is vital for alleviating neurodegenerative processes in AD. The formulation boosted the expression of Sirtuin-1 (SIRT1), a protein related to neuroprotection and longevity. Additionally, it lowered levels of miRNA-134, which is associated with neurodegenerative processes. This regulation encourages neurite outgrowth, improving neuronal connectivity and function.

## miRNA-106b

The levels of miRNA-106b were notably reduced in serum and brain tissues of both human and animal models of AD (Madadi et al., 2022; Liu et al., 2016; Kong et al., 2016). miR-106b-5p is one putative pleiotropic regulator of AD. Several studies have demonstrated the role of genes associated with autophagy in AD. Targeting the BECN1 gene, miR-106b5p, may have an impact on autophagy. The ubiquitin-proteasome system (UPS) is one protein degradation mechanism connected to the pathogenesis of AD. Numerous genes involved in the UPS system may be targets of miR-106b-5p. Furthermore, it has been demonstrated that miR-106b-5p impacts BBB transporters, indicating its involvement in multiple AD-related processes (Madadi et al., 2022). Thus, miR-106b-5p may play a significant role in various mechanisms contributing to the progression of AD. Treatment with resveratrol elevated the levels of miRNA-106b in a dose-dependent manner. The research by Kong et al. (2016) found that the 3′ untranslated region (3′UTR) of APP has a binding site for miRNA-106b. This indicates that miRNA-106b can directly influence APP expression, which is important because abnormal processing of APP is a key feature of AD. By increasing the levels of miRNA-106b, resveratrol may help lower the expression of target genes like APP, P62, and ApoA1, which are linked to inflammation and apoptosis in the context of AD (Kong et al., 2016).

## miRNA-146a

A key modulator of the inflammatory response, miRNA-146a is often increased in response to pro-inflammatory stimuli such as cytokines and pathogen-associated molecular patterns. Targeting IRAK-1’s mRNA lowers protein levels and lessens the inflammatory signaling pathways triggered by TLRs (Aslani et al., 2021). On the other hand, IRAK-1 plays a crucial role in the TLR signaling cascade by transmitting signals that trigger the production of pro-inflammatory cytokines and activate NF-Κb (Guo et al., 2024). Prolonged inflammatory reactions brought on by high IRAK-1 levels can be detrimental in neurodegenerative disorders. By blocking IRAK-1, the rise in miRNA-146a acts as a negative feedback mechanism to reduce excessive inflammation and shield neuronal cells from harm (Aslani et al., 2021).

In addition to its function with IRAK-1, miRNA-146a targets several genes implicated in inflammatory pathways, possibly including those linked to TSPAN12 (Negro-Demontel et al., 2024). TSPAN12, a tetraspanin family member, is crucial for immunological responses and neural development because it engages in cell adhesion, signaling, and interactions with other membrane proteins (Termini and Gillette, 2017). It has been connected to changes in the activation of microglia, immune cells in the brain that play a key role in neuroinflammatory reactions. MiRNA-146a probably affects TSPAN12 expression, which affects cell adhesion and communication in inflammatory situations, even if the precise connections between miRNA-146a and TSPAN12 are still being studied. Alterations in miRNA-146a and TSPAN12 expression may be involved in the deregulation of inflammatory responses in neurodegenerative illnesses such as AD, which can affect the health and function of neurons (Negro-Demontel et al., 2024). Furthermore, miRNA-146a can directly target the complement factor H (CFH) mRNA, resulting in decreased production of the CFH protein (Kong et al., 2016). CFH protein has an impact on the complement system and associated inflammatory reactions (Saxena et al., 2024). A crucial modulator of the complement system, CFH shields host cells from complement-mediated harm during inflammatory responses and inhibits overactivation that can harm tissue. Low levels of CFH are linked to increased neuroinflammation and neurodegeneration; CFH is necessary for preserving homeostasis in the brain and shielding neurons from inflammation-related harm (Negro-Demontel et al., 2024). The interaction between miRNA-146a and CFH creates a negative feedback loop, whereby increased miRNA-146a during inflammation leads to lower CFH levels, exacerbating inflammation. This cycle can raise miRNA-146a levels further, further reducing the expression of CFH (Kong et al., 2016). One possible therapeutic approach for reducing neuroinflammation and offering defense against neurodegenerative illnesses is to target the miRNA-146a/CFH axis. Restoring equilibrium in the inflammatory response may be facilitated by raising CFH levels or blocking miRNA-146a. Lukiw et al. (2008) discovered that miRNA-146a is upregulated in the brains of individuals with AD. This particular miRNA is affected by NF-κB signaling and is complementary to the 3′UTR of CFH, which is essential for modulating the brain’s inflammatory response. The rise in miRNA-146a levels is associated with a reduction in CFH expression in both AD brains and stressed cultured human neural (HN) cells exposed to factors such as interleukin-1 and amyloid beta. This suggests that increased levels of miRNA-146a lead to reduced CFH expression, which may exacerbate inflammation. The research also indicated that treating stressed HN cells with the NF-κB inhibitor pyrollidine dithiocarbamate or the resveratrol analog CAY10512 prevented the increase in miRNA-146a and helped restore CFH levels.

## Curcumin

Curcumin, a hydrophobic polyphenol with potent antioxidant, antibacterial, neuroprotective, anti-inflammatory, anti-cancer, antiproliferative, and immunomodulatory qualities, was isolated from the rhizome of *Curcuma longa* and is currently being explored as a possible drug for the treatment of neurological disorders such as AD (Genchi et al., 2024). Importantly, curcumin has the ability to control gene activation by influencing the expression of particular miRNAs (Hassan et al., 2019). This suggests a possible association between curcumin, AD, and miRNAs.

## miRNA-146a

The primary role of curcumin in treating AD and its connection to miR-146a’s involvement in the neuroinflammation process surrounding AD were the main topics of Gong et al.’s study (Gong and Sun, 2022). Participants were chosen from a pool of 20 people with AD and another 20 people who were matched in age and gender but did not have dementia or inflammation. Quantitative PCR with real-time technology was used to measure the levels of mir-146a in the peripheral vein and cerebral fluid serum and fluid samples. Researchers observed that plasma levels of miRNA-146a were notably reduced in the AD group compared to the control group. In contrast, the CSF levels of miRNA-146a were significantly elevated in the AD group, indicating a regulatory imbalance. Following the administration of low-dose curcumin to APP/PS1 mice, researchers observed a notable reduction in brain levels of miRNA-146a, suggesting that curcumin can influence the expression of this neuroinflammatory marker (Gong and Sun, 2022). Additionally, curcumin treatment led to significant decreases in Aβ and APP levels, indicating its potential to diminish the formation of amyloid plaques, a key characteristic of AD. The treatment also resulted in significant reductions in interleukin-1 beta (IL-1β) and inducible nitric oxide synthase (iNOS), which are markers linked to M1 microglial activation and inflammation. This suggests that curcumin effectively suppresses inflammatory signaling pathways. Conversely, the level of CFH, a molecule that helps regulate inflammation and encourage phagocytosis, markedly increased with curcumin treatment. In summary, these results indicate that curcumin diminishes neuroinflammation by decreasing miRNA-146a levels and inhibiting the M1 microglial phenotype. By increasing CFH expression and facilitating the clearance of Aβ, curcumin may help alleviate the inflammatory processes linked to AD.

## Quercetin

Quercetin is a flavonoid in tea, herbs, fruits, seeds, and nuts. Quercetin is recognized for its various established health benefits, including its protective effects against neurodegenerative diseases (Chiang et al., 2023; Goyal et al., 2024), mainly due to its ability to regulate miRNA expression, among other mechanisms (Ebrahimpour et al., 2020; Lv et al., 2018).

## miRNA-146a and miRNA-9

The study by Ebrahimpour et al. (2020) investigated how quercetin-conjugated superparamagnetic iron oxide nanoparticles (QCSPIONs) affect memory impairment caused by diabetes, focusing on inflammation-related miRNAs and the NF-κB signaling pathway in the hippocampus of diabetic rats. The findings indicated that diabetes notably increased the expression of several key apoptotic signaling molecules. Specifically, miRNA-146a and miRNA-9 are associated with inflammatory responses and neuronal cell death, while BACE1 and AβPP play roles in the production of Aβ, which is linked to the advancement of AD. Furthermore, Bax, a protein that promotes apoptosis, facilitates cell death, while Bcl-2 acts as a protective factor against apoptosis. QCSPIONs were more effective than pure quercetin in lowering the expression of miRNA-9, which is linked to inflammation and apoptosis. Additionally, QCSPIONs reduced the pathological activity of NF-κB, an important transcription factor that regulates inflammatory responses. This resulted in the normalization of BACE1 and AβPP levels, along with an improved Bax/Bcl-2 ratio, which is crucial for cell survival. Both quercetin and QCSPIONs were shown to reduce NF-κB activity, which is commonly elevated in inflammatory conditions. By regulating the levels of inflammatory miRNAs and inhibiting the NF-κB signaling pathway, quercetin—especially in its QCSPION form—demonstrated potential neuroprotective effects. This could help alleviate neuroinflammation and apoptosis linked to AD, thus improving memory function.

## miRNA-26a and miRNA-125b and miRNA-132

Quercetin has been found to significantly improve cognitive function in APP/PS1 mice with low vitamin D levels. The research conducted by Lv et al. (2018) indicated that quercetin, especially when administered alongside low vitamin D status (LVD), markedly enhanced cognitive performance in these mice, as demonstrated by their results in the Morris Water Maze test. Mice that received quercetin treatment demonstrated shorter times to reach the platform and a greater number of crossings in the target area. In the low vitamin D (LVD) group, quercetin treatment led to a significant decrease in Aβ plaques and phosphorylated tau (p-Tau) at Ser396 and Ser404. This is crucial because both Aβ accumulation and tau hyperphosphorylation are major pathological characteristics of AD that contribute to neuronal death and inflammation. Moreover, the study noted a reduction in neuroinflammation markers in the low vitamin D (LVD) group. Given that brain inflammation can worsen neurodegeneration, quercetin’s anti-inflammatory effects help alleviate this problem, promoting neuronal survival. In the hippocampus of the LVD group, quercetin treatment reduced the levels of miRNA-26a and miRNA-125b, both of which are linked to neuroinflammation and apoptosis. The decrease in miRNA-26a and miRNA-125b indicates a movement toward a more protective environment that lessens pro-apoptotic signals. In contrast, miRNA-132 levels increased, promoting neuroprotection and supporting cognitive function by enhancing synaptic plasticity and neuronal survival. By lowering the pro-apoptotic miRNAs while increasing the neuroprotective miRNA-132, quercetin helps create a balance that favors cell survival and diminishes inflammation.

## Epigallocatechin-3-Gallate (EGCG)

EGCG belongs to the class of flavonoids known as catechins, a subclass of polyphenols. A gallate group joined to epigallocatechin makes up its structure (Ciupei et al., 2024). The putative neuroprotective qualities of EGCG have drawn interest, especially in neurodegenerative illnesses like AD (Madhubala et al., 2024). EGCG might aid in shielding neurons from harm brought on by inflammation and oxidative stress (Madhubala et al., 2024). EGCG has been linked to the control of neuroinflammatory microRNAs, which can affect the brain’s inflammatory response.

## miR-34a-5p and miR-125b-5p

Aβ peptide synthesis, oxidative stress, chronic inflammation, and neuronal death in AD may all be influenced by the decrease of microglial miRNAs. Numerous investigations using animal models and AD patients have revealed downregulation of miR-34a-5p and miR-125b-5p. These alterations can be a reflection of early pathogenic events. Dysregulation of miR-34a-5p may lead to neuronal injury and cognitive impairment because it regulates genes linked to neuroinflammation and apoptosis. On the other hand, because miR-125b-5p is essential for synaptic function and neuronal survival, variations in its levels can significantly impact neuroinflammatory reactions and cognitive function. Serum samples from 27 AD patients in the study by Li et al. (2020) showed noticeably lower levels of miRNA-34a-5p and miRNA-125b-5p than those of healthy people. Primary mouse cortical neurons (MCN) and Neuro2a (N2a) cells treated with Aβ showed a similar decrease. Furthermore, it was discovered that under these circumstances, there was a rise in the expression of β-site BACE1, which is essential for forming Aβ. Reintroducing miRNA-34a-5p or miRNA-125b-5p into cells treated with Aβ significantly reduced oxidative stress and apoptosis, indicating that these miRNAs protect against Aβ-induced neurotoxicity. Both miRNAs were shown to target BACE1 directly, and the protective effects of miRNA-34a-5p and miRNA-125b-5p were lessened when BACE1 levels were restored. By increasing the expression of miRNA-34a-5p and miRNA-125b-5p in the study conducted by Li et al. (2020), EGCG may reduce BACE1 levels, leading to decreased Aβ accumulation and its associated neurotoxic effects. Moreover, EGCG aids in reducing apoptosis in neurons exposed to Aβ, which is crucial for preserving neuronal health and function in the context of AD.

## miRNA-125b, miRNA-9, and miRNA-191-5p

The protective effects of EGCG and the increased levels of miRNAs also help lower oxidative stress, contributing to neurodegeneration associated with AD. In the research conducted by Hong et al. (2017), EGCG was investigated for its potential therapeutic effects on AD. EGCG treatment in APP/PS1 transgenic mouse models showed improvements in cognitive abilities, as measured by the Morris water maze test. Numerous studies on EGCG have revealed that it may help treat AD by reducing cognitive impairment and improving learning capacity (Uchida et al., 2024; Sivamaruthi et al., 2024; Afzal et al., 2022). The research showed that EGCG treatment reversed the downregulation of circulating miRNAs (miRNA-125b, miRNA-9, and miRNA-191-5p) in AD models (Hong et al., 2017). Circulating miR-125b, miR-9, and miR-191-5p are downregulated in both animal models and human cases of AD (Hong et al., 2017; Kumar et al., 2013). miR-125b plays a role in controlling synaptic plasticity and neuronal survival. In neurodegenerative models, its expression levels can affect cognitive function (Hong et al., 2017). miR-125b plays a role in controlling synaptic plasticity and neuronal survival. In neurodegenerative models, its expression levels can affect cognitive function.

## Agathisflavone

Flavonoids and polyphenolic compounds found in plant species have various biological effects, such as antiviral, anticancer, anti-inflammatory, and antioxidant qualities (Chaves et al., 2022; Huang et al., 2023; Wang et al., 2012). The neuroprotective benefits of flavonoids may be related to their capacity to affect miRNA expression and their pro-inflammatory properties in microglial cells (Medrano-Jiménez et al., 2022). The flavonoid compound agathisflavone is present in various plants, particularly in the leaves of species such as agathis (e.g., the Kauri tree). Given its possible health advantages, especially regarding neuroprotection and anti-inflammatory actions, it has attracted interest (Dos Santos Souza et al., 2018). Agathisflavone is mainly associated with alterations in the inflammatory profile of microglia (Dos Santos et al., 2023). miRNAs significantly impact the development of neurodegenerative illnesses like AD because they are essential post-transcriptional regulators of gene expression in glial cells. They support the general health of neurons and the regulation of inflammatory responses.

## miRNA-146a and miRNA-155

The potential of agathisflavone, which is extracted from the leaves of Cenostigma pyramidal, to affect neuroinflammation in AD via controlling miRNAs in activated microglia was investigated in the study by Dos Santos et al. (2024). In this study, human C20 microglia were subjected to oligomers of Aβ and LPS to trigger an inflammatory response. After this exposure, the cells received treatment with agathisflavone to assess its effects. When human C20 microglial cells were activated with LPS or Aβ, the expression of miR-146a and miR-155 increased along with increases in IL-6, IL-1β, and NOS2, suggesting that miRNA-146a and miR-155 regulate the expression of mediators of inflammation. Treatment with agathisflavone (1 μM) led to a notable reduction in the levels of miRNA-146a and miRNA-155, along with the inflammatory cytokines IL-1β, IL-6, and NOS2. This suggests that agathisflavone possesses anti-inflammatory properties that can regulate the inflammatory response in microglia.

Furthermore, in Aβ-stimulated cells, an increase in phosphorylated STAT3 (p-STAT3), which is associated with inflammatory signaling, was observed. Treatment with agathisflavone resulted in a decrease in p-STAT3 levels, reinforcing its role in reducing inflammation. The study highlights the importance of miRNAs in the anti-inflammatory effects of agathisflavone. By downregulating pro-inflammatory miRNAs like miRNA-146a and miRNA-155, agathisflavone helps to mitigate the inflammatory response induced by Aβ and LPS. This modulation is essential for potentially protecting neurons from the detrimental effects of inflammation linked to AD.

## Osthole

Osthole, a coumarin derivative, exhibits multiple properties, such as anti-inflammatory, anti-apoptotic, and antioxidant effects. Studies suggest that Osthole has neuroprotective qualities, especially in maintaining neuronal synapses. Osthole can cross the BBB and exhibit considerable neuroprotective effects against AD, both *in vitro* and *in vivo*. However, the precise mechanisms behind these effects are not yet fully understood.

## miRNA-9

In the study by Li et al. (2016), a cell model of AD was created by introducing the APP695 Swedish mutant (APP695swe, APP) into mouse cortical neurons and human SH-SY5Y cells. Treatment with osthole was shown to improve cell viability and protect against cell death in cells overexpressing APP. Osthole treatment improved cell viability and protected against cell death in cells that overexpress APP. Furthermore, Osthole restored the amounts of vital synaptic proteins such as postsynaptic density-95 (PSD-95), synaptophysin, and synapsin-1. APP transduction has decreased the expression of PSD-95, SYP, and synapsin-1 in human cortical neurons and SH-SY5Y cells (Li et al., 2016). These neuroprotective effects were associated with an increase in the expression of miRNA-9, which is essential for mediating the effects of osthole. MiRNA-9 is expressed throughout neural development and adulthood, particularly in neurogenic brain areas. Researchers have recently begun looking into miRNA-9 decrease *in vitro* and *in vivo* (Che et al., 2014). The production of two proteins involved in apoptotic pathways, phosphorylated AMPKα (p-AMPKα) and CAMKK2, was reduced due to miRNA-9 overexpression (Li et al., 2016; Zhang et al., 2019). The activation of AMPK depends on CAMKK2 phosphorylating Thr-172. According to recent studies, Aβ oligomers may interfere with calcium homeostasis, leading to the loss of synapses and the activation of the CAMKK2-AMPK pathway, which could result in an influx of Ca^2+^ into cells (Mairet-Coello et al., 2013). Aβ42-induced synaptotoxicity can be prevented by miRNA-9 by blocking the CAMKK2-AMPK pathway (Mairet-Coello et al., 2013). This indicates that miRNA-9 might inhibit apoptosis and support synaptic integrity in the presence of Aβ.

## Naringin

Naringin (NR) is a flavanone glycoside comprising the flavanone naringenin and the disaccharide neohesperidose. It is an essential active ingredient in several traditional Chinese herbal medicines, such as Drynaria fortunei, *Citrus aurantium*, and *Citrus medica*. NR is also present in citrus fruits, contributing a bitter taste to their juices. It offers a variety of health benefits, including antioxidant, anti-inflammatory, anti-apoptotic, anti-ulcer, anti-osteoporotic, and anti-carcinogenic properties. NR has demonstrated beneficial effects on various CNS disorders, including AD. NR from Rhizoma drynariae has been shown to enhance cognition and lessen AD symptoms in animal models through miRNA regulation.

## miR-96-5p

In the study conducted by Wu et al. (2020) NR was recognized for its protective effects against the advancement of AD through various mechanisms. It was found to influence the expression of miRNA-96-5p, which negatively regulates Foxo1. miR-96-5p was reported to be highly expressed in AD. According to research, Foxo1 may act as a mediator between oxidative stress and AD (Paroni et al., 2014). It is believed that Foxo1 plays a key in preserving hippocampus neuronal homeostasis as people age, as evidenced by increased protein expression (Jenwitheesuk et al., 2017). After receiving NR therapy, the osteoblasts of AD mice showed a noticeable rise in Foxo1 expression and a significant decrease in miR-96-5p and Lcn2 expression. Lcn2, a protein linked to AD development, is inhibited by NR via modifying these levels. Lcn2 can cause neuroinflammation, contributing to AD development and course (Naudé et al., 2012). Combining NR treatment with the knockdown of miRNA-96-5p led to decreased oxidative stress markers. Specifically, NR boosted the levels of antioxidant enzymes such as superoxide dismutase. Additionally, NR treatment resulted in a significant reduction of Lcn2 levels, which is important because high levels of Lcn2 are linked to the progression of AD. Mice treated with NR demonstrated improvements in cognitive tests, indicating enhanced memory and learning abilities often impaired in AD.

## Future directions

To advance our understanding of the interplay between polyphenols and miRNAs in AD, it is crucial to develop robust preclinical *in vitro* and *in vivo* models before initiating clinical trials. These models will enable efficient screening of polyphenols and ensure that only the most promising candidates progress in the research pipeline, clarifying their pharmacokinetics and biological effects. Current preclinical models often fail to replicate AD pathology’s complexities, particularly the intricate interactions between inflammation, apoptosis, and Aβ accumulation. Advanced technologies such as 3D brain organoids and human-induced pluripotent stem cells (iPSCs) can provide deeper insights into how polyphenols affect miRNA regulation (Li et al., 2016). Future studies should also explore the specific mechanisms by which polyphenols modulate miRNA expression and their effects on neuroinflammatory and apoptotic pathways, which will aid in identifying potential therapeutic targets and enhance dietary interventions’ efficacy (Yan et al., 2022; Syarifah-Noratiqah et al., 2018). Furthermore, well-designed clinical trials are necessary to evaluate the therapeutic potential of polyphenol-rich diets or supplements in AD patients, focusing on cognitive outcomes and the modulation of miRNA expression related to neuroinflammation and synaptic integrity (Long et al., 2021; Colizzi, 2019). Investigating the synergistic effects of polyphenols alongside existing AD medications could improve treatment outcomes while minimizing side effects, leveraging the anti-inflammatory properties of polyphenols to enhance conventional therapies (Gong and Sun, 2022). Longitudinal research is essential to assess the long-term impacts of polyphenol consumption on cognitive decline and AD progression, identifying critical intervention periods (Lamport and Williams, 2021; Rosli et al., 2022). Finally, future investigations should focus on synthesizing and evaluating new polyphenol compounds with improved bioavailability and specificity for miRNA modulation, thereby enhancing their therapeutic potential in AD prevention (Liu et al., 2024; Aatif, 2023; El Monfalouti and Kartah, 2024; Elawad et al., 2024). By addressing these areas, researchers can uncover new strategies for managing AD and potentially improve patient outcomes through dietary and therapeutic interventions.

## Conclusion

The study highlights the significant role of polyphenols in mitigating apoptosis and neuroinflammation in AD through the regulation of miRNAs. Polyphenols have demonstrated potential in influencing Aβ metabolism and reducing inflammatory responses, which are crucial in AD progression. The intricate interaction between polyphenols and miRNAs suggests that these compounds could be promising therapeutic agents for managing AD. Abnormal miRNA expression is linked to key pathological features of the disease, emphasizing their importance as biomarkers and therapeutic targets.
